# Dissection of Mitochondrial Function via Chemical Perturbation and Single‐Cell Profiling

**DOI:** 10.1111/cpr.70216

**Published:** 2026-04-27

**Authors:** Hao Luo, Xiaona Yin, Hongxin He, Yaning Wang, Hongbo Zhang

**Affiliations:** ^1^ The Key Laboratory for Stem Cells and Tissue Engineering, Ministry of Education Zhongshan School of Medicine, Sun Yat‐sen University Guangzhou Guangdong China; ^2^ The SYSU‐YSG Joint Laboratory for Skin Health Research Sun Yat‐sen University Guangzhou Guangdong China; ^3^ State Key Laboratory of Pharmaceutical Biotechnology Nanjing University Nanjing Jiangsu China

**Keywords:** cell‐cycle regulation, chemical perturbation, mitochondrial function, mitochondrial stress signaling, single‐cell transcriptomics

## Abstract

Mitochondria play central roles in cellular energy metabolism and signal transduction, and maintenance of mitochondrial homeostasis is essential for proper cellular function. Rather than being regulated by individual genes alone, mitochondrial homeostasis is governed by coordinated functional modules, including glucose and lipid metabolism, the tricarboxylic acid (TCA) cycle, oxidative phosphorylation (OXPHOS), calcium handling, mitochondrial dynamics, mitochondrial reactive oxygen species (mtROS) regulation, and mitochondrial transcription and translation. However, how perturbation of these modules reshapes cellular states remains incompletely understood. Here, we combined targeted chemical perturbations with single‐cell RNA sequencing (scRNA‐seq) to systematically profile transcriptional responses to inhibition of core mitochondrial functional modules. Comparative analyses revealed both shared and module‐specific transcriptional programs, including recurrent co‐expression patterns across distinct perturbations. Analysis of mitochondrial gene expression across conditions implicated mtROS as an important regulator of mitochondrial respiratory chain (MRC) gene expression, potentially acting through activation of the mitochondrial integrated stress response (mtISR). Further comparative analysis of perturbations targeting individual MRC complexes uncovered distinct transcriptional and cellular consequences among complexes. Examination of cell‐cycle dynamics showed that mitochondrial perturbations generally suppress cell proliferation; inhibition of most MRC complexes was associated with G1‐phase arrest, whereas perturbation of complex III preferentially led to G2/M‐phase arrest, potentially reflecting differential engagement of p53‐associated signaling pathways. Finally, our analysis revealed both conserved and divergent transcriptional responses to mitochondrial perturbations between human and mouse cells. Together, these findings establish a systematic single‐cell framework for dissecting mitochondrial functional modules and highlight both shared and function‐specific principles by which mitochondrial perturbations influence cellular transcriptional states.

## Graphical abstract

We establish a systematic framework to dissect mitochondrial function at the module level by combining targeted chemical perturbations with scRNA‐seq. This approach reveals shared and module‐specific programs linking mitochondrial activity to mito‐nuclear communication, stress response, and cell cycle, highlighting the tight coupling between mitochondrial functional modules and cellular states.

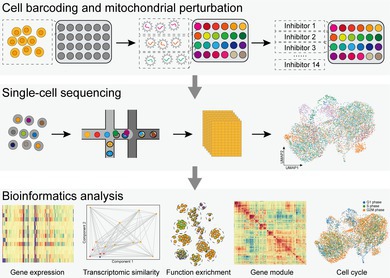

## 1 Introduction

Mitochondria have long been regarded as the powerhouse of eukaryotic cells [2]; however, extensive researches have revealed that their functions extend far beyond ATP production. Mitochondria serve as central hubs for cellular metabolism, biosynthesis, and signal transduction [3, 4, 5], thereby playing essential roles in the maintenance of cellular homeostasis [5]. Consequently, elucidating mitochondrial functions and their communication with the nucleus—particularly the regulation of nuclear gene expression—is critical for a comprehensive understanding of mitochondrial biology. Traditionally, mitochondrial function has been studied through a reductionist focus on individual genes or proteins. However, cellular functions are rarely executed by single proteins in isolation; instead, they emerge from the coordinated activity of functional modules composed of multiple interacting proteins [6]. How mitochondrial functional modules collectively regulate cellular states, and how their perturbation reshapes nuclear transcriptional programs, remain fundamentally important but incompletely addressed questions.

A wide array of mitochondria‐specific chemical inhibitors provides a powerful means to selectively perturb distinct mitochondrial functional modules and examine their consequences for cellular physiology. These inhibitors target diverse aspects of mitochondrial biology, including energy metabolism, mitochondrial reactive oxygen species (mtROS), mitochondrial dynamics, the mitochondrial central dogma, and intramitochondrial signaling [7, 8, 9, 10, 11]. Such approaches have enabled numerous foundational discoveries regarding mitochondrial function and its broader impact on cellular homeostasis [12, 13, 14, 15]. Nevertheless, systematic comparisons across multiple mitochondrial functional modules within a unified experimental framework remain limited.

In parallel, advances in omics technologies—including large‐scale transcriptomics and proteomics—have substantially expanded our understanding of protein function, subcellular localization, and functional organization within mitochondria [16, 17, 18, 19, 20]. However, most of these studies rely on bulk populations of cells or tissues, which can obscure cell‐to‐cell heterogeneity and mask distinct cellular responses to mitochondrial perturbations. Among available approaches, single‐cell transcriptomics offers a unique advantage by capturing transcriptional responses at both the systemic and individual‐cell levels, enabling a more comprehensive assessment of how mitochondrial function influences cellular states.

In this study, we present a high‐throughput and systematic strategy to interrogate mitochondrial functional modules by combining targeted chemical perturbations with single‐cell RNA sequencing (scRNA‐seq). We employed 14 mitochondria‐specific inhibitors targeting distinct functional modules, including glucose and lipid metabolism, the tricarboxylic acid (TCA) cycle, oxidative phosphorylation (OXPHOS), calcium homeostasis, mitochondrial dynamics, mtROS regulation, and mitochondrial transcription and translation. Cells subjected to different perturbations were distinguished using barcode‐based labeling, enabling joint analysis within a single scRNA‐seq dataset. Using this framework, we characterized transcriptional landscapes associated with distinct mitochondrial perturbations, analysed relationships among mitochondrial functional modules, and examined their connections to mitochondrial gene expression and cell‐cycle regulation. Finally, we compared mitochondrial functional responses between human and mouse cells. Together, this work provides a systematic framework for studying mitochondrial function from a modular and single‐cell perspective.

## 2 Results

### 2.1 Construction of Transcriptional Profiles for Mitochondrial Functional Perturbations

**FIGURE 1:**
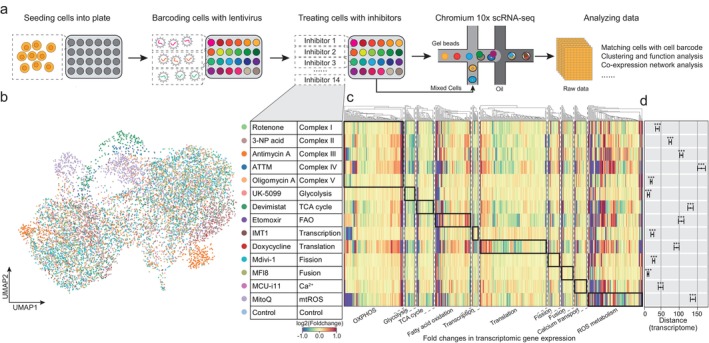
Overview of barcoded single‐cell perturbation of mitochondrial functional modules. (a) Schematic of barcoded single‐cell profiling. Cells were plated and labeled with lentiviral barcodes, followed by treatment with various inhibitors. Subsequently, the treated cells were subjected to scRNA‐seq to assess transcriptional changes. (b) UMAP visualization shows the distribution of cells following treatment with 14 different inhibitors. Each compound is indicated by a distinct colour, and the text next to each inhibitor denotes its primary target site. (c) Heatmap shows the transcriptomic changes of cells treated with different inhibitors. The vertical axis represents the different inhibitors, whereas the horizontal axis represents gene sets associated with various functions, which are collected from the GO database. The data represent fold changes relative to the control group. The black boxes highlight the corresponding inhibitors and their associated gene sets. (d) The figure shows the estimated distance of the transcriptome for each inhibitor perturbation group relative to the control, along with the 95% confidence intervals (CI). Statistical differences are indicated on the plot, ***p < 0.001 (Calculated by scDist).

To systematically perturb distinct mitochondrial functional modules, we selected 14 small‐molecule inhibitors targeting glucose and lipid metabolism, TCA cycle, OXPHOS, calcium homeostasis, mitochondrial dynamics, mtROS, and mitochondrial transcription and translation (Figure 1a) [21, 22]. We performed these experiments in C2C12 myoblasts, a well‐established model in which mitochondrial function is critical for maintaining and regulating cellular states [23, 24, 25]. All 14 compounds have previously been validated in this cell type or closely related systems [7, 12, 15, 26, 27, 28, 29, 30, 31, 32, 33, 34, 35]. We also evaluated the dosages used in this study by assessing cell proliferative capacity after 48 h of treatment with the 14 compounds. Most inhibitors reduced proliferation relative to the control group, while overall cell viability was largely preserved (Figure S1a). To enable parallel perturbation of multiple mitochondrial modules while unambiguously assigning each cell to its corresponding treatment, we combined cell barcoding with scRNA‐seq–based transcriptomic profiling (Figure 1a) [36]. Briefly, myoblasts were seeded into individual wells of a 24‐well plate and transduced with lentiviruses encoding unique 20‐nt barcodes. Barcoded cells were subsequently treated with one of the 14 mitochondrial inhibitors and pooled for scRNA‐seq. This strategy enabled simultaneous recovery of both transcriptome and barcode information, allowing precise identification of the perturbation applied to each individual cell. After quality filtering and data processing, a total of 9721 cells were retained; we generated a uniform manifold approximation and projection (UMAP) embedding of the single‐cell transcriptomes (Figure 1b). Data quality assessment revealed uniform distributions of read counts and detected genes across UMAP space and perturbation groups, with no evident outliers or major batch effects (Figure S1b–f).

Most mitochondrial perturbations occupied distinct regions of the UMAP space relative to control cells, reflecting treatment‐associated transcriptional changes (Figure 1b and Figure S2). Among the 14 compounds, antimycin A, ammonium tetrathiomolybdate (ATTM), devimistat, etomoxir, and mitoquinone (MitoQ) produced the most pronounced separation from controls, indicating substantial transcriptomic remodeling. In contrast, treatments with IMT1, MCU‐i11, MFI8, mitochondrial division inhibitor 1 (Mdivi‐1), oligomycin A, rotenone, and UK‐5099 resulted in comparatively subtler shifts in global transcriptomic profiles (Figure S2). To assess whether perturbations with weaker global effects nonetheless elicited relevant transcriptional responses, we examined expression changes within Gene Ontology (GO) gene sets corresponding to the targeted mitochondrial processes [37, 38]. Indeed, each perturbation was associated with significant changes in at least one functionally related gene set (Figure 1c). Moreover, global differential expression analysis revealed that all perturbations produced statistically significant transcriptomic changes relative to controls (Figure 1d), confirming that each compound exerted measurable transcriptional effects on the cells.

Clustering of single‐cell transcriptomes using the Leiden algorithm revealed that cells exposed to strongly acting inhibitors often segregated into two main subclusters (Figure S3a,b). For example, antimycin A‐treated cells were distributed across clusters 11 and 14, ATTM‐ and devimistat‐treated cells across clusters 17 and 19, and MitoQ‐treated cells across clusters 10 and 13. Reducing the clustering resolution collapsed these subclusters into two major groups, with cells from each perturbation distributed between them (Figure S3c,d). Control cells were similarly divided, with 60.2% assigned to group 0 and the remainder to group 1, and other treatment groups showing comparable distributions (49%–67%) between the two groups (Figure S3d). To interpret the biological basis of this dichotomous separation, we examined gene expression signatures characteristic of each group (Figure S3e). Group 0 was enriched for markers of activated myoblasts, including *Myod1* and members of the MEF2 family, whereas group 1 exhibited transcriptional signatures of proliferation and a more undifferentiated state, marked by expression of *Mki67* (Figure S3f). These results indicate that the two clusters primarily reflect intrinsic cellular heterogeneity, likely arising from spontaneous activation and early differentiation in culture. Consistent with this interpretation, cells subjected to the same perturbation but assigned to different groups displayed high overall transcriptional similarity (Figure S3g). Trajectory analysis showed that the two groups lay along a differentiation path, with all perturbations symmetrically distributed and no perturbation‐induced branch (Figure S3h–j). Cells in group 0 within each perturbation group consistently upregulated differentiation‐related programs (Figure S3k).

Together, these analyses establish a single‐cell transcriptomic atlas of chemical perturbations targeting core mitochondrial functional modules, providing a foundation for systematically exploring how mitochondrial functions interact with global cellular gene expression programs across distinct cellular states.

### 2.2 Systematic Analysis of Functional Similarities, Differences, and Gene Modules Across Mitochondrial Modules

**FIGURE 2:**
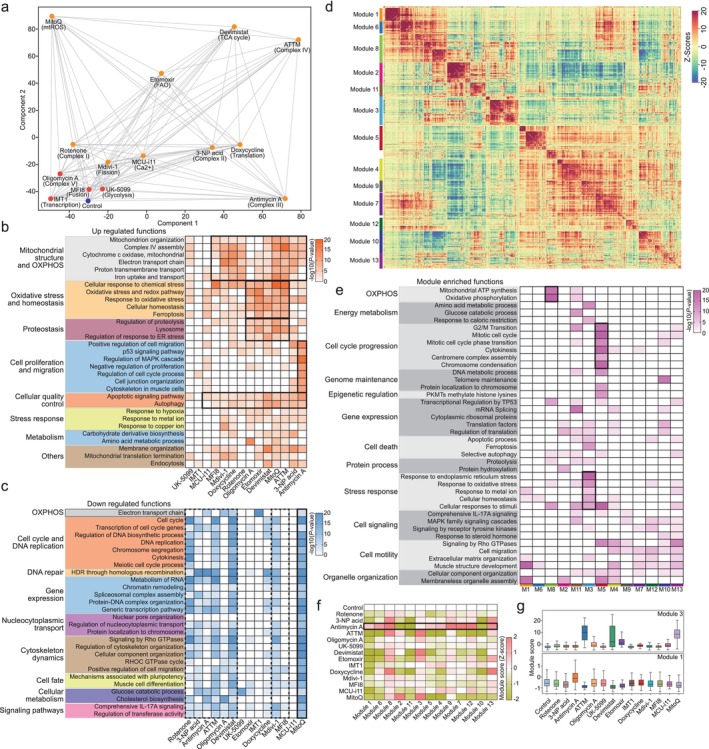
Functional analysis of single‐cell transcriptomes under mitochondrial modules perturbation. (a) The plot shows the relative distances between different perturbation groups after MDS dimensionality reduction, where closer points indicate higher transcriptomic similarity. Blue points represent the control group, and red points indicate those closer to the control group. (b, c) Heatmaps show the functional enrichment of upregulated and downregulated genes in different perturbation groups. The vertical axis displays functional categories, with colour blocks distinguishing the main functional categories, and the category names are shown next to the colour blocks. The horizontal axis represents the different inhibitor treatment groups. Black boxes highlight functions that are commonly enriched across certain treatment groups. (d) Heatmap shows gene expression modules identified using Hotspot, with successfully detected modules indicated by different colours on the left; a total of 13 modules were identified. (e) Heatmap shows the functional enrichment of genes corresponding to different modules. The vertical axis distinguishes functional categories, with category names displayed next to the colour blocks. The horizontal axis represents different gene modules. Black boxes highlight the positions of the functions associated with Module 3, 5, and 8. (f) Heatmap shows the enrichment of different gene modules in different treatment groups after *Z*‐Score transformation. Red indicates positive enrichment, while green indicates negative enrichment. Black boxes highlight the enrichment pattern of antimycin A across different gene modules. (g) Boxplot shows the distribution of Module 1 and 3 scores across cells grouped by treatment conditions.

We first assessed transcriptional similarities among cells subjected to different mitochondrial perturbations (Figure 2a). Consistent with the separation observed in UMAP space, inhibitors targeting most mitochondrial respiratory chain (MRC) complexes, the TCA cycle, fatty acid oxidation (FAO), and mtROS induced pronounced transcriptional divergence from control cells. In contrast, several perturbations—including UK‐5099, an inhibitor of mitochondrial pyruvate transport in glucose metabolism [39]—elicited relatively modest transcriptomic changes. This observation is consistent with previous reports that glutamine oxidation can partially compensate for reduced pyruvate availability [40]. Similarly, MFI8 and Mdivi‐1, which perturb mitochondrial fusion and fission, respectively, resulted in comparatively mild global transcriptional alterations, consistent with their primary effects on mitochondrial morphology and dynamics rather than broad transcriptional regulation [41]. Notably, perturbations targeting the five OXPHOS complexes exhibited relatively low transcriptional similarity to one another (Figure 2a), suggesting that individual complexes exert distinct regulatory influences on cellular transcriptional programs.

To further characterize relationships among mitochondrial functional modules, we identified differentially expressed genes (DEGs) for each perturbation relative to control and performed functional enrichment analysis based on these DEGs. Upregulated functions were predominantly associated with core cellular processes, including OXPHOS, stress responses, cellular homeostasis, and proteostasis. Importantly, these functions exhibited preferential enrichment under specific perturbations rather than uniform activation across all conditions (Figure 2b and Figure S4). Functions related to OXPHOS were enriched across many perturbations, particularly those targeting MRC complexes, the TCA cycle, mitochondrial dynamics, FAO, mitochondrial translation, and mtROS. In addition, oxidative stress‐ and proteostasis‐related functions were more prominently enriched following perturbation of MRC complexes IV and V, FAO, the TCA cycle, and mtROS [42]. Although the involvement of the TCA cycle and OXPHOS in cellular stress responses is well established, the induction of oxidative stress–related genes by MitoQ may reflect its reported capacity to disrupt redox balance under certain conditions. Indeed, MitoQ has been shown to enhance oxidative stress via quinone redox cycling and superoxide generation under high oxygen exposure [43, 44]. Whether similar mechanisms operate in myoblasts remains to be determined. Notably, functions related to cell proliferation and migration were preferentially enriched under perturbation of MRC complex III, indicating a potential complex‐specific regulatory role.

We next examined downregulated genes and their associated functions (Figure 2c and Figure S5). These functions encompassed OXPHOS, cell‐cycle progression, DNA repair, gene expression, nucleocytoplasmic transport, cytoskeletal organization, and cellular metabolism. Perturbations that directly impaired mitochondrial energy production—including inhibition of the five MRC complexes and the TCA cycle—resulted in widespread downregulation across these functional categories. In contrast, perturbation of mitochondrial dynamics yielded more distinct effects: inhibition of mitochondrial fusion (MFI8) produced relatively fewer transcriptional changes than inhibition of fission (Mdivi‐1), consistent with the notion that fusion may act as a protective or compensatory response under stress [41]. Inhibition of mtROS also resulted in substantial downregulation of multiple cellular functions. Although UK‐5099 showed fewer overall enriched functional categories, it nevertheless induced downregulation of glucose metabolism‐related genes (Figure 2c and Figure S5), confirming its functional activity.

To further delineate shared and distinct transcriptional responses across perturbations, we applied Hotspot analysis to identify co‐regulated gene expression modules [45]. This analysis identified 13 gene modules, which were subsequently annotated based on associated cellular functions (Figure 2d,e). These modules could also be mapped to specific perturbation groups, thereby linking chemical inhibition of mitochondrial functions to shared cellular response programs (Figure 2f). The identified modules encompassed diverse functions, including energy metabolism, cell‐cycle regulation, epigenetic control, stress responses, cell death, and organelle organization (Figure 2e). Several modules displayed broad activation across perturbations. For example, Module 3 (stress response) and Module 8 (OXPHOS‐related genes) were upregulated in most treatment groups (Figure 2f), suggesting that they may represent core transcriptional responses associated with mitochondrial perturbation. In contrast, Module 5, enriched for cell‐cycle‐related genes, was suppressed in the majority of perturbations (Figure 2f), consistent with the close relationship between mitochondrial function and cell‐cycle regulation [46]. Further analysis revealed that perturbations of MRC complex IV, the TCA cycle, FAO, and mtROS led to particularly strong co‐upregulation of Module 3 (Figure 2g), indicating that these functional modules may converge on shared mitochondrial stress‐response pathways. Module 1, associated with the organization of membraneless organelles, was preferentially enriched under perturbation of MRC complex III, suggesting a potential link between complex III activity and regulation of phase‐separated cellular structures. Notably, antimycin A‐mediated inhibition of MRC complex III induced broad upregulation across multiple gene modules (Figure 2f), consistent with its pronounced impact on mitochondrial stress and redox signaling, although the underlying mechanisms warrant further investigation.

Together, these analyses provide a systematic view of how perturbation of distinct mitochondrial functional modules reshapes nuclear transcriptional programs through both shared and module‐specific gene expression responses.

### 2.3 Mitochondrial Stress‐Induced Remodelling of Mitochondrial Gene Expression

Given the robust self‐regulatory capacity of mitochondria [47, 48], we next sought to determine how mitochondrial perturbations reshape mitochondrial gene expression programs. Here, we define mitochondrial genes as protein‐coding genes that are either encoded by the mitochondrial genome or encoded in the nucleus and targeted to mitochondria, as annotated in the MitoCarta3.0 database [18].

**FIGURE 3:**
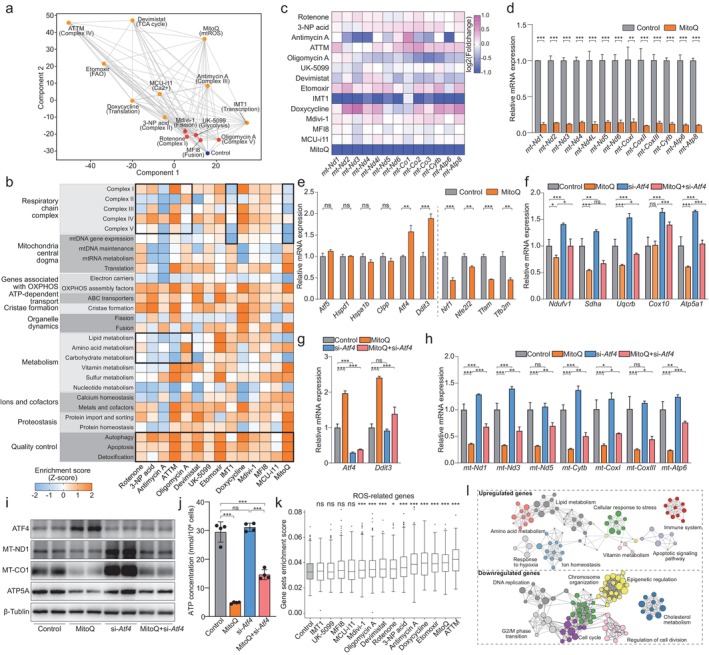
Changes in nuclear‐ and mitochondria‐encoded genes under different perturbations. (a) The plot shows the relative distances between the transcriptomes of mitochondrial‐related genes across different perturbation groups after MDS dimensionality reduction, where closer points indicate higher transcriptomic similarity. Blue points represent the control group, and red points indicate those closer to the control group. (b) AUCell enrichment scores were calculated for mitochondrial gene sets annotated in the MitoCarta3.0 database across different perturbation groups. Enrichment scores for each gene set were *Z*‐Score normalized to control across perturbations. The heatmap displays mitochondrial functional gene sets (rows) and perturbation groups (columns). Gene sets were grouped into major functional categories, indicated by coloured blocks and labeled alongside the heatmap. Orange and blue colours denote positive and negative enrichment, respectively. Black boxes highlight gene sets discussed in the main text. (c) Heatmap shows log_2_‐transformed fold changes of the 13 mitochondrial DNA‐encoded genes across different perturbation groups relative to control. Rows represent genes and columns represent perturbations. (d, e) qPCR analysis shows expression changes of the indicated genes after MitoQ treatment. Data are presented as mean ± SD. ns not significant, **p < 0.01, ***p < 0.001 (two‐tailed Student's *t*‐test). (f–h) qPCR analysis shows expression changes of the indicated genes after MitoQ treatment, *Atf4* knockdown, or both. Data are presented as mean ± SD. ns not significant, *p < 0.05, **p < 0.01, ***p < 0.001 (two‐way ANOVA test). (i) Protein level of ATF4, MT‐ND1, MT‐CO1, and ATP5A in C2C12 upon MitoQ treatment, *Atf4* knockdown, or both. (j) Intracellular ATP levels were measured under MitoQ treatment, *Atf*4 knockdown, or their combination. The vertical axis represents ATP concentration normalized per 10^6^ cells. ns not significant, ***p < 0.001 (two‐way ANOVA test). (k) Boxplot shows AUCell scores of ROS‐related gene sets from GO database across cells, grouped by perturbation. ns not significant, *p < 0.05, ***p < 0.001 (one‐way ANOVA test). (l) The visualization depicts functional enrichment of upregulated and downregulated genes in the MitoQ‐treated group relative to control, with different colours indicating distinct functional categories.

We first constructed a transcriptomic similarity map based on the expression of nuclear‐encoded mitochondrial genes across all single cells (Figure 3a). In general, perturbations that produced strong or weak global transcriptional effects showed corresponding impacts on mitochondrial gene expression. One notable exception was MCU‐i11, an inhibitor of the mitochondrial calcium uniporter (MCU) complex, which exhibited a disproportionately strong effect on mitochondrial gene expression relative to its global transcriptomic impact. Consistent with this observation, previous studies have reported similar downregulation of mitochondrial genes following genetic disruption of MCU [49], supporting a specific role for mitochondrial calcium signaling in regulating mitochondrial gene expression.

To further characterize mitochondrial transcriptional remodeling, we classified mitochondrial gene sets according to MitoCarta3.0 functional categories and calculated relative enrichment scores between treated and control cells with AUCell (Figure 3b). The annotated mitochondrial genes spanned 28 functional categories, including MRC complexes, mitochondrial central dogma, cristae organization, mitochondrial dynamics, metabolism, ion and protein homeostasis, and quality control. Across perturbations, mitochondrial genes were predominantly upregulated, with particularly consistent induction of quality control‐related programs, including apoptosis, autophagy, and detoxification pathways. This pattern was observed across all 14 perturbations, indicating that even relatively mild mitochondrial stress elicits a broad and conserved mitochondrial stress response [50, 51, 52]. Despite this global trend, perturbations also produced category‐specific effects. In particular, inhibition of individual MRC complexes resulted in heterogeneous transcriptional responses among OXPHOS‐related genes (Figure 3b), highlighting functional divergence among the complexes. In contrast, genes involved in carbohydrate, lipid, and amino acid metabolism showed a general tendency toward downregulation following inhibition of the five MRC complexes, with the notable exception of complex V. In addition, we observed that perturbation of mtROS using MitoQ, similar to inhibition of mitochondrial transcription by IMT1, led to downregulation of mitochondrial OXPHOS genes (Figure 3b).

Because 13 core OXPHOS subunit genes are located in the mitochondrial genome [19, 53], we next examined the expression of these mitochondrial‐encoded genes across all perturbations (Figure 3c). Strikingly, MitoQ treatment resulted in a pronounced and coordinated downregulation of all 13 mitochondrial‐encoded OXPHOS genes. We validated this observation experimentally using quantitative real‐time PCR (qPCR), which confirmed that MitoQ treatment significantly reduced the expression of both mitochondrial‐encoded and nuclear‐encoded MRC genes (Figure 3d and Figure S6a). To determine whether this effect was specific to MitoQ, we treated cells with another mtROS scavenger, visomitin [54], and observed similar coordinated suppression of mitochondrial and nuclear MRC gene expression (Figure S6b,c). These findings suggest synchronized regulation of mitochondrial and nuclear genomes following mtROS perturbation [55].

Previous studies have reported that MitoQ can modulate mitochondrial stress signaling and coordinate mitochondrial‐nuclear gene expression [56]. To investigate whether mitochondrial stress‐response pathways were involved, we examined the expression of genes associated with the mitochondrial unfolded protein response (mtUPR) and the mitochondrial integrated stress response (mtISR). We observed significant upregulation of *Atf4* and *Ddit3* (Figure 3e and Figure S6d), key transcriptional regulators of the mtISR, whereas expression of canonical mtUPR components, including *Hspd1*, *Hspa1b*, and *Clpp*, remained unchanged. In parallel, the expression of transcriptional regulators controlling nuclear‐encoded mitochondrial genes (*Nrf1*) and mitochondrial genome expression (*Tfam* and *Tfb2m*) was increased.

Therefore, we knocked down *Atf4* in the presence of MitoQ treatment and found that MRC‐related genes were significantly upregulated compared with the MitoQ‐treated group (Figure 3f–h). Consistent changes were also observed at the protein level (Figure 3i). We finally measured intracellular ATP levels and found that MitoQ treatment decreased ATP, whereas subsequent *Atf4* knockdown partially restored ATP levels (Figure 3j). Together, these results support a model in which mtROS perturbation induces coordinated mitochondrial gene regulation primarily through activation of the mtISR. Moreover, this phenomenon is not limited to C2C12 cells. Analysis of publicly available datasets showed that MitoQ treatment led to downregulation of mitochondrial‐encoded genes in two human breast cancer cell lines, MDA‐MB‐231 and SKBR3, with broader suppression of nuclear‐encoded OXPHOS genes in SKBR3 cells (Figure S6e).

Consistent with the central role of ROS in cellular homeostasis, inhibition of all mitochondrial functional modules altered the expression of mtROS‐related genes (Figure 3k). To further characterize the transcriptional consequences of mtROS perturbation, we performed functional enrichment analysis of DEGs following mtROS inhibition (Figure 3l). Genes involved in stress responses, ion homeostasis, hypoxia adaptation, apoptotic signaling, vitamin and nutrient metabolism, and major metabolic pathways—including carbohydrate, lipid, and amino acid metabolism—were broadly upregulated. In contrast, genes associated with epigenetic regulation, chromosome organization, DNA replication, cholesterol metabolism, and cell‐cycle progression were consistently downregulated, reflecting coordinated suppression of nuclear regulatory programs and proliferative capacity. Together, these findings highlight mtROS as a key mediator of mitochondrial‐nuclear communication and a central regulator of cellular homeostasis [57].

### 2.4 Functional Similarities and Differences Among Respiratory Chain Complexes

**FIGURE 4:**
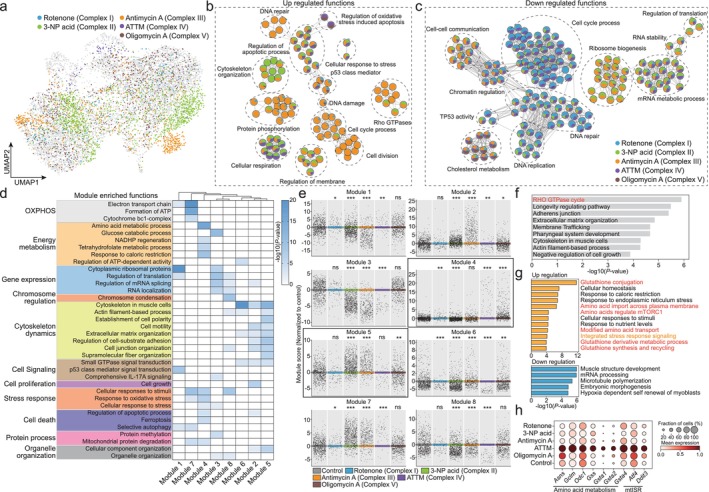
Analysis of mitochondrial respiratory chain complex functions and gene expression modules. (a) UMAP plot shows the distribution of cells treated with inhibitors targeting the five MRC complexes. (b, c) Visualization of functional enrichment of genes upregulated or downregulated in each MRC‐related treatment group relative to the control, with different colours representing distinct perturbations. (d) Heatmap shows functional enrichment across different gene modules. Enriched functional terms on the rows are grouped into major functional categories indicated by coloured blocks, with category labels shown alongside. Gene modules on the columns were hierarchically clustered. (e) Distribution of module scores across treatment groups associated with the five MRC complexes. Module scores for each perturbation group were normalized by subtracting the mean score of the control group. Distinct colours represent different perturbation groups, and black boxes highlight Module 4 and 5. ns not significant, *p < 0.05, **p < 0.01, ***p < 0.001 (one‐way ANOVA test). (f) Functional enrichment analysis was performed using genes co‐upregulated with Module 5 and exhibiting a correlation coefficient *r* greater than 0.9 with the module score. (g) Functional enrichment analysis was performed using genes co‐upregulated with Module 4 and exhibiting a correlation coefficient *r* greater than 0.9 or less than −0.9 with the module score. Red denotes amino acid metabolism‐related pathways, whereas orange denotes stress response‐related pathways. (h) Dotplot shows the expression levels of genes involved in amino acid metabolism and the mtISR across different MRC perturbation groups.

Given the functional divergence observed among the five MRC complexes, we next examined their distinct transcriptional consequences in greater detail. Cells treated with inhibitors targeting each of the five complexes occupied largely non‐overlapping regions in UMAP space, indicating complex‐specific transcriptional responses (Figure 4a). To quantify the extent of divergence among complexes, we compared DEGs induced by inhibition of each complex and analysed both shared and unique functional programs. Among upregulated functional categories (Figure 4b), inhibition of complexes II and III elicited the most pronounced responses, with strong enrichment of functions related to protein phosphorylation, cell‐cycle progression and division, apoptosis, oxidative stress responses, DNA damage repair, GTPase‐mediated signaling, and cytoskeletal organization. In contrast, among downregulated functions (Figure 4c), inhibition of complexes II and III was associated with suppression of pathways involved in cellular communication as well as gene transcription and translation. Inhibition of complexes I, IV, and V also produced substantial transcriptional changes, with downregulated functions converging primarily on cell‐cycle regulation, chromatin organization, DNA replication and repair, and cholesterol metabolism. These observations indicate that while all MRC complexes influence core cellular programs, individual complexes exert distinct regulatory effects on specific biological processes.

To further characterize gene expression patterns and their functional organization following MRC perturbation, we performed Hotspot analysis using cells from the five complex‐inhibition conditions. This analysis identified eight co‐regulated gene expression modules (Figure 4d). These modules were associated with diverse biological functions, including oxidative phosphorylation (Module 7), energy metabolism (Module 4), gene expression (Module 3), chromatin regulation (Module 8), cytoskeletal dynamics (Module 5), stress responses (Module 4), and cellular quality control (Module 4 and 7), while other modules displayed more mixed functional characteristics. Mapping these gene expression modules to individual MRC perturbations revealed that inhibition of complexes II and III induced particularly strong and coordinated transcriptional changes across multiple modules (Figure 4e). Specifically, genes in Module 1 and 3 were coordinately downregulated, whereas genes in Module 2, 5, and 7 were upregulated. To explore whether these coordinated responses reflect shared regulatory mechanisms, we focused on Module 5, which was strongly associated with cytoskeletal dynamics. Functional enrichment analysis of genes highly correlated with Module 5 (correlation coefficient *r* > 0.9) revealed significant enrichment of GTPase cycle‐related pathways (Figure 4f), consistent with the established role of GTPase signaling in regulating cytoskeletal organization [34, 58]. These results suggest that inhibition of complexes II and III may converge on cytoskeletal regulation through shared GTPase‐mediated signaling pathways.

In contrast, inhibition of complex IV (ATTM treatment) preferentially activated Module 4, which is enriched for energy metabolism‐related genes (Figure 4e). Functional enrichment analysis of genes co‐upregulated within Module 4 revealed prominent alterations in amino acid metabolism pathways, accompanied by activation of mtISR‐associated signaling (Figure 4g). Consistent with this interpretation, expression analysis of key regulators of amino acid metabolism and mtISR pathways supported activation of this response following complex IV inhibition (Figure 4h).

### 2.5 Connections Between Mitochondrial Functions and Cell‐Cycle Dynamics

Mitochondria are tightly linked to cell‐cycle regulation [46], and our analyses consistently indicated that perturbation of distinct mitochondrial functional modules alters cell‐cycle‐related transcriptional programs (Figures 2c and 4b,c). We therefore sought to systematically investigate how disruption of individual mitochondrial functions influences specific aspects of cell‐cycle regulation.

**FIGURE 5:**
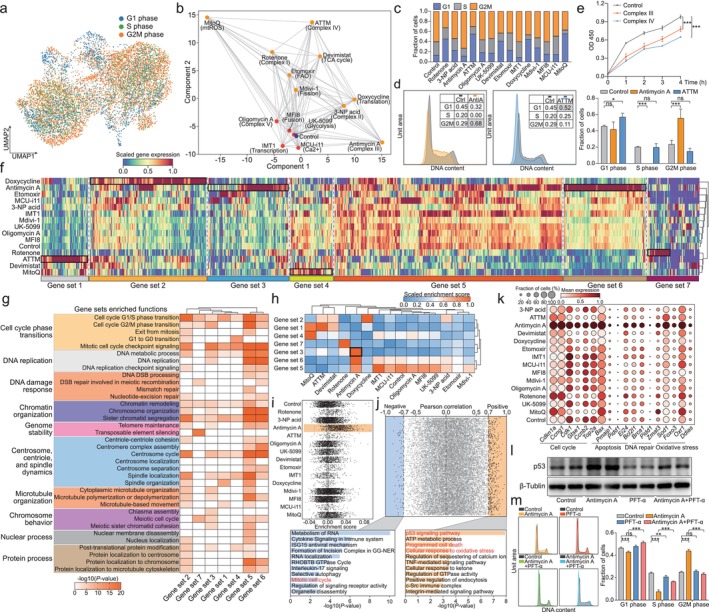
Altered cell‐cycle progression following perturbation of distinct mitochondrial functional modules. (a) UMAP plot shows the distribution of cells in G1‐, S‐, and G2/M‐ phases based on cell‐cycle scoring in Scanpy. (b) The plot shows the relative distances between the transcriptomes of cell‐cycle‐related genes across different perturbation groups after MDS dimensionality reduction. Blue points represent the control group, and red points indicate those closer to the control group. (c) Stacked bar plot shows the proportion of cells in each cell‐cycle phase across different perturbation groups. (d) Flow cytometry analysis of cell‐cycle phase distribution following treatment with antimycin A and ATTM. Cell‐cycle modelling was performed using FlowJo. Data are presented as mean ± SD. ns not significant, *p < 0.05, ***p < 0.001 (one‐way ANOVA test). (e) Cell proliferation was measured by CCK‐8 assay at OD 450 after antimycin A (Complex III) and ATTM (Complex IV) treatment. ***p < 0.001 (two‐way ANOVA test). (f) Heatmap displaying the expression of cell cycle‐related genes collected from GO database across perturbation groups. Normalized expression values were used for hierarchical clustering of both rows and columns. Based on expression patterns, genes were grouped into seven gene sets, indicated at the bottom. Black boxes highlight gene sets that show high expression under specific treatments. (g) The heatmap shows functional enrichment across different gene sets. Columns represent enriched functional terms, grouped into major functional categories indicated by coloured blocks, with category names shown alongside. Rows represent the different gene sets. (h) The heatmap shows the enrichment of different gene sets across perturbation groups. Black boxes highlight the location of gene set 3 enrichment under antimycin A treatment. (i) The plot shows the distribution of enrichment scores for gene set 3 across perturbation groups, with scores normalized relative to the control group. The antimycin A group is highlighted. (j) Scatter plot shows all genes co‐expressed with gene set 3. Genes with *r* > 0.7 (positively correlated) are shown in orange, and genes with *r* < −0.7 (negatively correlated) are shown in blue. Functional enrichment results are shown separately for positively and negatively correlated genes. The red highlights pathways related to the cell cycle, p53, and oxidative stress. (k) Dotplot shows the expression of genes related to cell‐cycle and apoptosis pathways across different perturbation groups. (l) Protein level of p53 in C2C12 upon antimycin A treatment, PFT‐*α* treatment, or both. (m) Flow cytometry analysis of cell‐cycle phase distribution after treatment with antimycin A, PFT‐*α*, or both. Data are presented as mean ± SD. ns not significant, **p < 0.01, ***p < 0.001 (two‐way ANOVA test).

We first inferred the cell‐cycle state of each cell using Scanpy‐based scoring (Figure 5a) and calculated transcriptomic similarity among perturbation groups using a curated set of 1011 cell‐cycle‐associated genes from the GO database (Figure 5b). Most perturbations induced pronounced changes in cell‐cycle‐related gene expression, whereas treatment with oligomycin A, MFI8, UK‐5099, IMT1, and MCU‐i11 produced comparatively modest effects. Consistent with these transcriptomic patterns, cell‐cycle phase distributions revealed that perturbation groups exhibiting transcriptional profiles closer to controls also displayed similar cell‐cycle compositions (Figure 5c). Notably, while several mitochondrial perturbations increased the proportion of cells in the G1‐phase, antimycin A treatment resulted in a marked accumulation of cells in the G2/M‐phase. Flow cytometric cell‐cycle analysis independently confirmed these observations, demonstrating that Antimycin A increased the G2/M‐phase population, whereas ATTM treatment led to an increased fraction of cells in the G1‐phase (Figure 5d). Despite arrest at distinct cell‐cycle stages, both Antimycin A and ATTM treatments significantly suppressed cell proliferation (Figure 5e), indicating that disruption of different mitochondrial functions can converge on growth inhibition through distinct cell‐cycle checkpoints.

To determine whether these cell‐cycle alterations arise from shared or distinct regulatory programs, we analysed the expression patterns of the 1011 cell‐cycle‐associated genes across all perturbation groups. This analysis identified seven distinct gene‐expression patterns, which we clustered into seven gene sets (Figure 5f). Functional annotation revealed that these gene sets collectively spanned diverse cell‐cycle‐related processes, including phase transitions, genome stability maintenance, DNA replication and repair, chromatin regulation, and nuclear organization (Figure 5g). Mapping these gene sets to individual perturbations revealed distinct associations between mitochondrial functions and specific cell‐cycle programs (Figure 5h). Gene set 5 was broadly expressed across multiple groups—including control, IMT1, MCU‐i11, oligomycin A, MFI8, UK‐5099, 3‐NP acid, etomoxir, and Mdivi‐1—and was enriched for core cell‐cycle processes, suggesting that it represents a housekeeping cell‐cycle program. Consistently, these groups exhibited similar cell‐cycle phase distributions (Figure 5c). In contrast, perturbations associated with elevated expression of gene sets 1, 2, 4, and 7 predominantly exhibited G1‐phase arrest, indicating that mitochondrial dysfunction can induce coordinated suppression of proliferative programs through broad transcriptional remodeling.

We next sought to understand why perturbation of MRC complex III specifically induced G2/M‐phase arrest. Gene set 3 was selectively enriched following complex III inhibition (Figure 5h,i). Functional enrichment analysis of genes positively correlated with gene set 3 (correlation coefficient *r* > 0.7) revealed significant enrichment of p53‐associated signaling pathways (Figure 5j). Consistent with previous studies demonstrating that p53 activation can induce G2/M arrest through p21‐mediated mechanisms [59], we observed increased expression of *Cdkn1a* and other p53 downstream targets involved in apoptosis, DNA damage responses, and oxidative stress following antimycin A treatment (Figure 5k). Compared with other perturbations, antimycin A induced the strongest activation of p53‐associated transcriptional programs. To further validate this, we treated antimycin A‐exposed cells with the p53 inhibitor pifithrin‐α (PFT‐α). At the protein level, PFT‐α treatment reduced p53 levels compared with the antimycin A group (Figure 5l). Flow cytometric analysis further confirmed that inhibition of p53 rescued the G2/M‐phase arrest induced by antimycin A (Figure 5m).

Together, these results demonstrate that mitochondrial functional modules exert both shared and complex‐specific control over cell‐cycle dynamics in myoblasts, with distinct mitochondrial perturbations engaging different transcriptional programs to regulate cell‐cycle arrest and proliferative capacity.

### 2.6 Conserved and Divergent Transcriptomic Responses to Mitochondrial Functional Module Perturbations in Human and Mouse Cells

**FIGURE 6:**
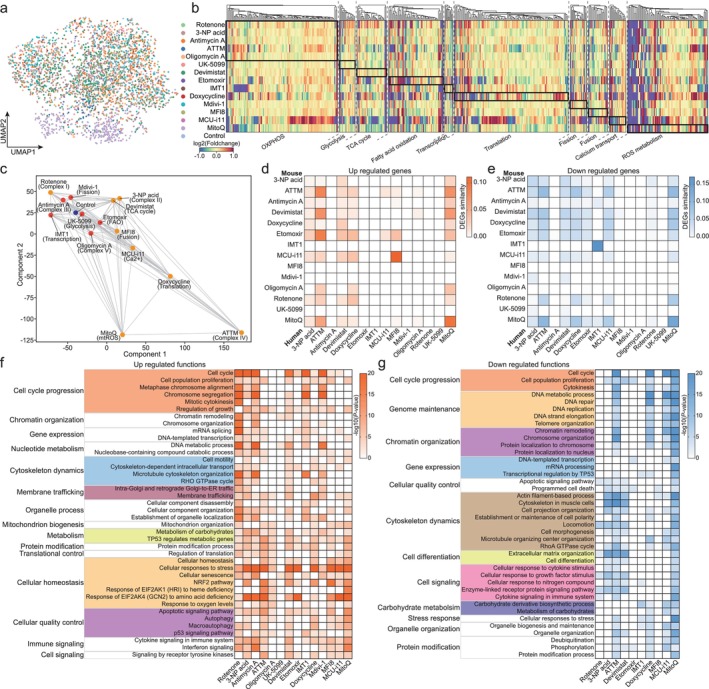
Comparison of human and mouse cell responses to mitochondrial functional modules perturbations. (a) UMAP visualization of single‐cell transcriptomes following treatment with 14 inhibitors, with each compound coloured distinctly. (b) Heatmap shows the transcriptomic changes of cells treated with different inhibitors. The vertical axis represents the different inhibitors, whereas the horizontal axis represents gene sets associated with various functions, which are collected from the GO database. The data represent fold changes relative to the control group. (c) The plot shows the relative distances between different perturbation groups after MDS dimensionality reduction, where closer points indicate higher transcriptomic similarity. Blue points represent the control group, and red points indicate those closer to the control group. (d, e) Heatmaps show the consistency of DEGs within the same perturbation in human and mouse for upregulated (left) and downregulated (right) genes, calculated using the Jaccard similarity index. (f, g) Heatmaps show the functional enrichment of upregulated and downregulated genes in different perturbation groups. The vertical axis displays functional categories, with colour blocks distinguishing the main functional categories, and the category names are shown next to the colour blocks; the horizontal axis represents different perturbation groups. Highlighted colour blocks indicate functional terms similar to those observed in mouse data.

To examine the conservation and divergence of mitochondrial functional responses across cell types, we applied the same experimental and analytical workflow to primary human myoblasts. Cells were barcoded, treated with the corresponding mitochondrial inhibitors, and subjected to scRNA‐seq, followed by integrated transcriptomic analysis (Figure 6a), yielding a total of 3191 cells for downstream analysis. We assessed the data quality and found that the distributions of read counts and detected genes were also uniform in the sequencing data from primary human cells (Figure S7a,b).

We first examined transcriptional changes induced by perturbation of different mitochondrial functional modules. Consistent with observations in mouse myoblasts, distinct perturbations elicited distinct transcriptional responses in human cells (Figure 6b). Using whole‐transcriptome profiles, we calculated transcriptional distances between perturbation groups and controls (Figure 6c). Similar to the mouse dataset, perturbations with ATTM, MitoQ, and doxycycline produced pronounced transcriptional divergence, whereas oligomycin A, UK‐5099, and IMT1 clustered closer to the control group. In addition, etomoxir, Mdivi‐1, and antimycin A also exhibited relatively modest transcriptomic shifts in human myoblasts compared with controls.

To directly compare gene‐level responses between species, we quantified the overlap of DEGs induced by each perturbation using the Jaccard similarity index. Overall, DEG overlap between human and mouse ranged from 0% to 15%, indicating limited gene‐level conservation (Figure 6d,e). Notably, downregulated DEGs showed greater cross‐species consistency than upregulated DEGs (Figure 6e). Among all perturbations, inhibition of MRC complex IV (ATTM) and mtROS (MitoQ) exhibited relatively higher similarity between species in both upregulated and downregulated gene sets, suggesting more conserved regulatory responses for these mitochondrial functions. Comparison of the transcriptomic profiles between these two cell types revealed only mild similarity across different treatments (Figure S7c).

Because different species may employ distinct gene sets to regulate similar biological processes, we next performed functional enrichment analysis of DEGs following mitochondrial perturbations. The functions enriched among upregulated genes in human cells largely overlapped with those observed in mouse cells, including pathways related to cell proliferation, cytoskeletal organization, cellular homeostasis, quality control, metabolism, and membrane transport (Figure 6f). Perturbation of the mitochondrial respiratory chain, the TCA cycle, and mtROS consistently resulted in widespread functional disruption across species. Stress response pathways were also broadly enriched across multiple perturbation groups. Analysis of downregulated DEGs revealed similar pathway‐level concordance between species (Figure 6g). As observed in mouse myoblasts, downregulated functions in human cells were enriched for pathways related to the cell cycle, chromatin regulation, gene expression, cytoskeletal organization, cell differentiation, and carbohydrate metabolism. Notably, MitoQ treatment in human cells produced broad transcriptional effects, including coordinated downregulation of all mitochondria‐encoded genes and widespread suppression of nuclear‐encoded respiratory chain complex genes (Figure S7d,e). In contrast, perturbations with etomoxir, IMT1, and MFI8 affected a more limited set of functional pathways. Unlike in mouse myoblasts, treatment with antimycin A, oligomycin A, UK‐5099, and Mdivi‐1 did not yield significant enrichment of downregulated pathways in human cells, suggesting specific differences in transcriptional sensitivity to certain mitochondrial perturbations.

Taken together, these results indicate that although mitochondrial perturbations elicit substantial gene‐level divergence between human and mouse cells, the core functional responses at the pathway level are largely conserved.

## 3 Discussion

Although individual mitochondrial genes and pathways have been extensively studied [22, 60], a systematic framework for interrogating mitochondrial functional modules and their global cellular consequences has remained limited. Here, we aimed to establish an experimental strategy that enables functional dissection of mitochondria at the modular level. With the expanding availability of chemical inhibitors targeting specific mitochondrial proteins and pathways [61, 62], it has become increasingly feasible to perturb mitochondrial biology in a controlled and functionally defined manner. Accordingly, we selected 14 widely used inhibitors targeting major mitochondrial functional modules and applied them to systematically interrogate mitochondrial function and its downstream transcriptional consequences.

To enable multiplexed profiling of these perturbations, we adopted a cell‐barcoding strategy inspired by the ChemPerturb‐seq framework [36]. By combining engineered barcode integration with the 10× Genomics single‐cell RNA‐seq platform, we established a scalable chemical perturbation‐based single‐cell profiling approach. This strategy allowed us to directly compare transcriptional responses across multiple mitochondrial perturbations within a unified experimental and analytical framework. Using this approach, we generated single‐cell transcriptomes from cells treated with all inhibitors. Comparative analysis revealed both shared and perturbation‐specific transcriptional programs, enabling us to map downstream cellular responses to distinct mitochondrial functional disruptions. Perturbation of core mitochondrial processes—particularly the MRC, lipid metabolism, and the TCA cycle—elicited widespread cellular changes [63, 64, 65]. Notably, we observed that many perturbations induced broad upregulation of functional programs, whereas downregulated genes were more consistently associated with cell‐cycle‐related pathways. This pattern is consistent with compensatory stress responses, whereby cells activate alternative pathways to maintain essential functions following mitochondrial impairment [66, 67, 68]. Nevertheless, despite such compensatory activation, perturbation of mitochondrial redox balance still resulted in impaired cell proliferation, underscoring the essential role of mitochondrial function in sustaining cellular growth [69].

By focusing on the relationship between MRC perturbation and cell‐cycle regulation, we found that inhibition of most MRC complexes induced G1‐phase arrest, consistent with previous studies [70, 71, 72]. In contrast, inhibition of complex III using antimycin A led to pronounced accumulation of cells in the G2/M‐phase. Similar observations have been reported elsewhere [73], although the underlying mechanisms remain incompletely understood. Our data further indicate that inhibition of different MRC complexes elicits distinct transcriptional and functional outcomes, with complex III perturbation exerting particularly strong and broad effects. One potential explanation is that complex III plays a central role in the assembly and stability of respiratory supercomplexes [74], structures critical for efficient electron transport and energy metabolism. In addition, complex III uniquely generates reactive oxygen species (ROS) on both sides of the inner mitochondrial membrane, positioning it as a key node in redox‐dependent mito‐nuclear signaling. Consistent with this idea, mtROS‐related genes were broadly represented across multiple regulatory modules in our dataset, highlighting the pervasive influence of ROS in mitochondrial and cellular regulation [75, 76].

Beyond global transcriptomic changes, we examined how mitochondrial perturbations reshape mitochondrial gene expression itself, a critical aspect of mitochondrial adaptation to stress. Across most perturbations, mitochondrial genes exhibited a general tendency toward upregulation, consistent with prior reports showing that mitochondrial stress activates transcription of nuclear‐encoded mitochondrial chaperones, proteases, and respiratory components to restore organelle function [47, 66]. In contrast, treatment with the mtROS scavengers MitoQ and visomitin resulted in pronounced downregulation of MRC‐related genes encoded by both the nuclear and mitochondrial genomes, a phenotype resembling that observed upon inhibition of mitochondrial translation by IMT1. Previous studies have demonstrated coordinated regulation between nuclear‐ and mitochondrial‐encoded genes [55]; however, the observation that mtROS suppression also disrupts this coordination suggests additional regulatory layers that remain to be elucidated.

Mechanistically, we observed activation of the mtISR, whereas canonical markers of the mtUPR remained largely unchanged. This finding is consistent with reports showing that mtISR activation can suppress respiratory chain gene expression [77]. Notably, most prior studies have described mtISR activation in the context of elevated mtROS [77, 78, 79], whereas scenarios in which mtROS levels decrease while mtISR remains activated have been reported less frequently. Our data therefore suggest that mtISR activation may not strictly require elevated mtROS, highlighting a more complex relationship between mitochondrial redox state and stress signaling. Given that both excessive and insufficient mtROS can disrupt cellular homeostasis—high mtROS promoting DNA damage, apoptosis, and senescence [80, 81], and low mtROS impairing essential redox homeostasis [76]—mtROS likely functions as a tightly regulated signaling molecule with dual roles [82]. We speculate that physiological levels of mtROS may be required to sustain proper expression of MRC‐related genes, although this hypothesis warrants further experimental validation.

Mitochondria exhibit heterogeneity, from subcellular spatial differences to variability across cell types [83, 84]. Which features are shared across mitochondria and which are condition‐specific remains to be fully elucidated. In this study, we focused on myogenic cells; however, mitochondria are also highly abundant in tissues such as the heart, liver, and brain. Whether similar phenomena are observed in these tissues, and how mitochondrial features differ between mitochondria‐rich and mitochondria‐poor regions represent important questions for future investigation.

Murine experiments were performed in C2C12 cells, an immortalized myoblast cell line with robust proliferative and differentiation capacity, whereas cross‐species comparisons used primary human myoblasts, which more closely reflect the in vivo state. Although many conserved functional features were observed, future validation in primary murine myogenic cells will be needed.

Finally, our study has several limitations. Although the inhibitors used here effectively target distinct mitochondrial functions, many exhibit pleiotropic effects, including induction of apoptosis, changes in mitochondrial membrane potential, or altered ROS production [9, 85, 86]. Future studies would benefit from the development or application of more selective compounds to minimize such confounding effects. In addition, the cellular effects of the inhibitor displayed a pronounced dose‐dependent pattern. For example, previous studies have shown that NADH consumption progressively decreases with increasing rotenone concentration [87]. Therefore, we currently lack a unified framework to quantitatively compare the relative strength of different perturbations, as cellular responses can vary substantially depending on dose and exposure time [87, 88]. Moreover, the availability of agonists that selectively activate specific mitochondrial pathways would further enhance functional interrogation and enable more precise dissection of mitochondrial regulatory mechanisms. And further analysis of inhibitor‐treated cells at the level of cellular subpopulations will also be needed.

## 4 Materials and Methods

### 4.1 Cell Culture

The murine myoblast cell line C2C12 was cultured in high‐glucose DMEM (10‐013‐CVRC, Corning) supplemented with 10% fetal bovine serum (FBS) (FSP500, ExCell Bio). For primary human myoblasts, cells were derived from specimens previously isolated in our laboratory during earlier studies, which had received approval from the institutional ethics committee [89, 90]. No new human‐derived samples were obtained for the present study. The isolation and culture of primary human myoblasts were performed according to our laboratory's previously published procedures [90]. Both cell types were cultured in a humidified atmosphere at 37°C supplemented with 5% CO_2_. Cells were dissociated using 0.25% Trypsin–EDTA (25200072, Gibco), and centrifugation was carried out at 300 g for 5 min.

### 4.2 Cell Barcoding

To label different cell populations with distinct barcodes, we designed 15 unique 20‐nt cellular barcodes corresponding to each inhibitor treatment and control (sequences listed in Table S1) based on non‐targeting sequences derived from the GeCKO v2 library [91]. Each barcode was individually cloned into the lentiGuide‐Puro plasmid via the BsmBI restriction sites, and sequence integrity was verified by Sanger sequencing [91]. For lentiviral production, each barcode‐containing plasmid was co‐transfected with psPAX2 and pMD2.G plasmids into 293T cells using X‐tremeGENE HP DNA Transfection Reagent (06366236001, Roche), according to the manufacturer's instructions. After 8 h of transfection, the medium was replaced, and lentiviral supernatants were collected at 48 h. The supernatants were centrifuged at 1500 g for 5 min and filtered through a 0.22‐μm filter. The resulting lentiviruses were then used to infect the target cells, followed by 2 μg/mL puromycin (0219453925, MP Bio) selection for 48 h to obtain successfully transduced populations. Following selection, cells were maintained in standard culture medium without puromycin and subjected to subsequent inhibitor treatments.

### 4.3 Perturbation of Barcoded Cells With Inhibitors and scRNA‐Seq

For the barcode‐positive cells, each barcode‐defined population was treated with a different inhibitor, with a total of 14 inhibitors tested, including rotenone (HY‐B1756, MedChemExpress), Mdivi‐1 (HY‐15886, MedChemExpress), UK‐5099 (HY‐15475, MedChemExpress), devimistat (HY‐15453, MedChemExpress), ammonium tetrathiomolybdate (HY‐W076067, MedChemExpress), 3‐nitropropanoic acid (HY‐W012875, MedChemExpress), oligomycin A (HY‐16589, MedChemExpress), IMT1 (HY‐134539, MedChemExpress), etomoxir (HY‐50202, MedChemExpress), MCU‐i11 (HY‐W194810, MedChemExpress), MFI8 (HY‐150031, MedChemExpress), doxycycline (HY‐N0565, MedChemExpress), MitoQ (HY‐100116A, MedChemExpress), antimycin A (MS0070‐10MG, MKBio). The optimal working concentrations for each inhibitor were determined based on previously published studies (details and references are provided in Table S2). After 48 h of treatment, the cells were dissociated using trypsin and resuspended in PBS containing 0.04% BSA to generate a single‐cell suspension for scRNA‐seq. We utilized the Chromium Next GEM Single Cell 5′ Reagent Kits v2 (PN‐1000263, 10× Genomics) to prepare single‐cell libraries following the manufacturer's instructions. Library preparation consisted of two parts: the transcriptome library and the cell barcode library. The cell barcode library was generated using the 5′ CRISPR Kit (PN‐1000451, 10× Genomics) with the manufacturer's instructions. The library was sequenced by Novogene (Guangzhou, China).

### 4.4 scRNA‐Seq Data Process

The raw reads were processed with Cell Ranger (version 7.1.0) to generate gene expression profiles and assign cell barcodes. Raw reads were mapped to GRCh38 for human cells (available at: http://cf.10xgenomics.com/supp/cell‐exp/refdata‐cellranger‐GRCh38‐3.0.0.tar.gz) and mm10‐2020‐A for mouse cells (available at: https://cf.10xgenomics.com/supp/cell‐exp/refdata‐gex‐mm10‐2020‐A.tar.gz). Data preprocessing, including normalization, log transformation, detection of highly variable genes, regression of cell cycle and technical variance (the same procedure was applied to both mouse and human samples, and all subsequent analyses were conducted using the regressed data), principal component analysis (PCA), and UMAP visualization, were performed using Scanpy (version 1.9.6) with default parameters [92]. *t*‐test was applied to identify DEGs in each perturbation group as compared to the control using sc.tl.rank_gene_groups. Genes with an adjusted *p* value < 0.01 were considered differentially expressed.

### 4.5 Functional Enrichment

Functional enrichment analysis was performed based on the identified differentially expressed genes using the online tool Metascape (https://metascape.org) [93]. Cytoscape (version 3.10.4) was used to visualize functional enrichment results based on the GONetwork files generated by Metascape. Because single‐group and multi‐group analyses are calculated slightly differently in Metascape, single‐group comparisons tend to yield more pathways, as smaller gene sets are not filtered out, whereas multi‐group analyses highlight pathways that are shared across groups or show differences between groups [93].

### 4.6 Gene Sets Scoring

Gene set enrichment scores were calculated using AUCell (version 1.22.0) [94]. AUCell first ranks all expressed genes per cell using the AUCell_buildRankings function, and then computes the area under the curve (AUC) for each input gene set using the AUCell_calcAUC function. This AUC value represents the enrichment score, and each cell was assigned one score per gene set.

### 4.7 Gene Module Identification

Gene modules were identified using the Hotspot (version 1.0) [45]. Gene modules were derived using the Hotspot create_modules function, which clusters genes based on pairwise autocorrelation patterns on the cell–cell *k*‐nearest neighbour (kNN) graph. Each resulting module represents a group of genes exhibiting coordinated variation across the similarity graph. Default parameters were used unless otherwise specified.

### 4.8 Transcriptome Similarity Calculation

Transcriptomic alterations upon perturbation with different inhibitors, relative to both the control group and other perturbation groups, were assessed using scDist (version 1.1.5) [95]. scDist estimates the transcriptomic distances between cell populations and provides statistical calculations. Using this tool, we computed the distances between all perturbation groups and applied multidimensional scaling (MDS) for dimensionality reduction [96], projecting the distances between different perturbation groups onto a two‐dimensional plane. The relative distances between perturbation groups were visualized using a scatter plot.

### 4.9 Cross‐Species Transcriptome Similarity Analysis

Similarity analyses of transcriptional profile across mouse and human were performed in R (version 4.3.1) using the MetaNeighbor package (version 1.22.0) [97]. Human and mouse datasets were converted to SingleCellExperiment objects for R analysis, retaining only orthologous genes. Unsupervised cross‐dataset similarity was then assessed using MetaNeighborUS function with all orthologous genes and default parameters. The output is an area under the receiver operating curve (AUROC) matrix, in which higher AUROC values indicate greater transcriptional similarity across species under the same inhibitor conditions. The results were visualized as a heatmap.

### 4.10 Cellular Differentiation Trajectory Analysis

To investigate whether cellular heterogeneity was induced by the perturbation, trajectory analysis was performed using Monocle (version 2.38.0) [98]. After estimation of size factors and dispersion parameters, genes detected in at least 10 cells were retained, and cells with exceptionally high total read counts were excluded. Genes for trajectory inference were selected using the differentialGeneTest function, and genes with *q* < 0.01 were considered. After dimensionality reduction using the DDRTree function, cells were ordered along the inferred trajectory with the orderCells function. The plot_cell_trajectory function was used to visualize the results.

### 4.11 RNA Isolation and Quantitative PCR

The RNA extraction was performed using the Trizol reagent (15596026, Invitrogen) following the manufacturer's instructions. A total of 500 ng of RNA was reverse transcribed into cDNA using the PrimerScript RT Master Mix (RR036A, TaKaRa). Quantitative PCR was performed using the PerfectStart Green qPCR SuperMix (AQ601, TransGen Biotech) according to the manufacturer's instructions for gene quantification. Relative mRNA expression of each gene was calculated using the 2^−ΔΔ^CT method. All primers used in this study are listed in Table S3.

### 4.12 Flow Cytometry Analysis

Flow cytometry analyses were performed with a flow cytometer (Beckman Coulter) and analysed with FlowJo (version 10) software. Briefly, cells were dissociated and centrifuged to remove the supernatant, followed by fixation in 1 mL of pre‐chilled 70% ethanol at 4°C overnight. Cells were then centrifuged at 1000 g for 5 min to remove the ethanol and washed once with ice‐cold PBS. Subsequently, cells were incubated with RNase A (R8020, Solarbio) at 37°C for 30 min. DNA was stained with propidium iodide (P8080, Solarbio) at 4°C for 30 min, then analysed by flow cytometry.

### 4.13 Protein Extraction and Western Blot

Protein samples were extracted using RIPA lysis buffer (FD008, Fude). After lysis on ice for 10 min, the lysates were centrifuged at 12,000 g for 10 min at 4°C, and the supernatants were collected for protein quantification using a BCA assay kit (P0012S, Beyotime). Subsequently, loading buffer (P0015L, Beyotime) was added to the supernatants. For mitochondrial complex proteins, samples were incubated at 37°C for 30 min without boiling, whereas the remaining samples were denatured at 100°C for 5 min. All samples were then subjected to SDS‐PAGE. The following antibodies were used for western blot analysis: anti‐MT‐ND1 (ab181848, Abcam), anti‐MT‐CO1 (55159T, Cell Signaling Technology), anti‐ATP5A (ab14748, Abcam), anti‐ATF4 (11815T, Cell Signaling Technology), anti‐p53 (2524T, Cell Signaling Technology), anti‐β‐Tublin (TA‐10, ZSGB‐BIO), anti‐Mouse IgG (115‐035‐003, Jackson ImmunoResearch), anti‐Rabbit IgG (111‐035‐003, Jackson ImmunoResearch).

### 4.14 RNA Interference‐Mediated Knockdown

*Atf4* knockdown was performed using small interfering RNA (siRNA). Cells were transfected with siRNA targeting *Atf4* using Lipofectamine RNAiMAX, following the manufacturer's instructions. The final concentration of siRNA was 50 nM. After 8 h of transfection, the medium was replaced with fresh complete culture medium. The siRNA sequence is as follow: GCTGCTTACATTACTCTAA. The siRNAs were synthesized by RiboBio, and the control siRNA sequence was provided by the RiboBio.

### 4.15 ATP Concentration Detection

The detection of ATP concentration is performed using ATP Assay Kit (ab83355, Abcam) according to the reagent instructions.

### 4.16 Statistics

All bioinformatic analyses related to scRNA‐seq data processing, as well as the number of biological replicates, statistical methods, and significance for all plots, are detailed in the Methods and corresponding figure legends.

## Author Contributions

**Hao Luo:** conception of the work, performing experiments, data analysis and interpretation, drafting the article, revision of the article. **Xiaona Yin:** conception of the work, data analysis and interpretation, drafting the article, revision of the article. **Hongxin He** and **Yaning Wang:** performing experiments, partial data analysis, revision of the article. **Hongbo Zhang:** conception of the work, overall project supervision, revision of the article. All authors read and approved the final manuscript.

## Funding

This work was supported by Ministry of Science and Technology of the People's Republic of China (2022YFA1104900, 2024ZD0530500), National Natural Science Foundation of China (W2511023, 31871370), Open Fund of State Key Laboratory of Pharmaceutical Biotechnology, Nanjing University, China (KF‐202503), Guangzhou Science and Technology Planning Project (2024A04J4614), Fundamental Research Funds for the Central Universities (24ykzy002).

## Conflicts of Interest

The authors declare no conflicts of interest.

## Supporting information


**Figure S1:** Quality control of single‐cell data following different inhibitor treatments. (a) The line graph shows the results of the CCK8 assay measuring cell viability/proliferative capacity after 48 h of treatment with 14 inhibitors. Data are presented as mean ± SD. (b, c) UMAP plot shows the read counts and gene counts for each cell, with darker colours indicating higher values. (d) UMAP visualization shows the distribution of cells from three different batches, with each batch represented by a distinct colour. (e, f) Boxplot shows the distribution of read counts and gene counts across different treatment groups.
**Figure S2:** UMAP plot shows the distribution of cells from 14 perturbation groups and control in mouse myoblasts, with each treatment highlighted in orange.
**Figure S3:** Analysis of cellular heterogeneity. (a) UMAP plot shows the clustering results obtained using the Leiden algorithm. (b) Stacked bar plot shows the distribution of different treatment groups across each cluster. The horizontal axis represents the proportion, and the vertical axis represents the clusters. (c) UMAP plot of myoblast cells shows heterogeneity, with two distinct clusters. (d) Stacked bar plot shows the distribution of cells from each perturbation across Group 0 and Group 1. (e) Functional enrichment analysis of Group 0 and Group 1 based on genes upregulated in one group versus the other. (f) UMAP plot shows the expression levels of *Myod1*, *Mki67*, *Mef2a*, and *Mef2c* across cells, with darker colours indicating higher expression. The violin plots on the right provide a further statistical comparison of the expression of the genes shown on the left between the two groups. Solid lines indicate the mean, and dashed lines indicate the quartiles. *** *p* < 0.001 (two‐tailed Student's *t*‐test). (g) Dendrogram of hierarchical clustering shows the transcriptomic similarity of cells from each perturbation located in Group 0 and Group 1. Cells from Group 0 and Group 1 are shown in blue and orange, respectively. Cells within each group were z‐normalized relative to the control cells in the corresponding group [1]. (h–j) Cell trajectory analysis was performed using Monocle and visualized by group 0 and group 1, pseudotime, and perturbation labels. (k) Functional enrichment analysis of multiple gene sets. The heatmap shows enriched functions of group 0‐upregulated genes among the DEGs between group 0 and group 1 in cells treated with each inhibitor.
**Figure S4:** Functional enrichment of genes upregulated in response to each mitochondrial inhibitor compared with control. Functional enrichment results were generated using the top 20 pathways automatically identified by Metascape for each treatment versus control.
**Figure S5:** Functional enrichment of genes downregulated in response to each mitochondrial inhibitor compared with control. Functional enrichment results were generated using the top 20 pathways automatically identified by Metascape for each treatment versus control.
**Figure S6:** Expression of mitochondrial genes after treatment with mitochondrial ROS scavengers. (a) Expression of nuclear‐encoded respiratory chain complex genes following MitoQ treatment, shown as a bar plot. Data are presented as mean ± SD. *p < 0.05, **p < 0.01, ***p < 0.001 (two‐tailed Student's *t*‐test). (b, c) Expression of mitochondria‐encoded and nuclear‐encoded respiratory chain complex genes following visomitin treatment, shown as a bar plot. Data are presented as mean ± SD. ns not significant, **p < 0.01, ***p < 0.001 (two‐tailed Student's *t*‐test). (d) Expression of stress response genes following visomitin treatment, shown as a bar plot. Data are presented as mean ± SD. ns not significant, ***p < 0.001 (two‐tailed Student's *t*‐test). (e) Heatmap shows analysis of the GSE148111 dataset, including two cell lines (MDA‐MB‐231 and SKBR3). The expression changes of the indicated genes under MitoQ treatment are displayed along the horizontal axis for each cell line. Data were normalized to transcript per million (TPM) and scaled for visualization.
**Figure S7:** Transcriptome data quality and gene expression across different perturbation groups in human data. (a, b) UMAP plot shows the read counts and gene counts for each cell, with darker colours indicating higher values. Boxplot of right panel shows the distribution of read counts and gene counts across different treatment groups. (c) Heatmap shows similarity of transcriptomic profiles between human and mouse cells under the same treatment, as assessed by MetaNeighbor. Values represent AUROC scores. (d) Heatmap shows log_2_‐transformed fold changes of the 13 mitochondrial DNA‐encoded genes across different perturbation groups relative to control. Rows represent genes and columns represent perturbations. (e) Heatmap shows log_2_‐transformed fold changes of nuclear‐encoded MRC genes across different perturbation groups relative to control. Rows represent genes and columns represent perturbations.

**Table S1:** Inhibitors and corresponding cell barcodes.

**Table S2:** Inhibitors, corresponding working concentrations, and references.

**Table S3:** Primers used in qPCR.

## Data Availability

All raw sequencing data reported in this paper have been deposited to Genome Sequence Archive in National Genomics Data Center at https://ngdc.cncb.ac.cn/gsa/ and accession codes are HRA017352 and CRA040203. The dataset GSE148111 was obtained from the NCBI Gene Expression Omnibus database.
