# Supplementary material for: Dissection of Mitochondrial Function via Chemical Perturbation and Single‐Cell Profiling
Source: Cell Prolif. 2026 Apr 27;59(9):e70216. doi: 10.1111/cpr.70216 (PMC13325944; doi:10.1111/cpr.70216)

Supplementary Figure 1

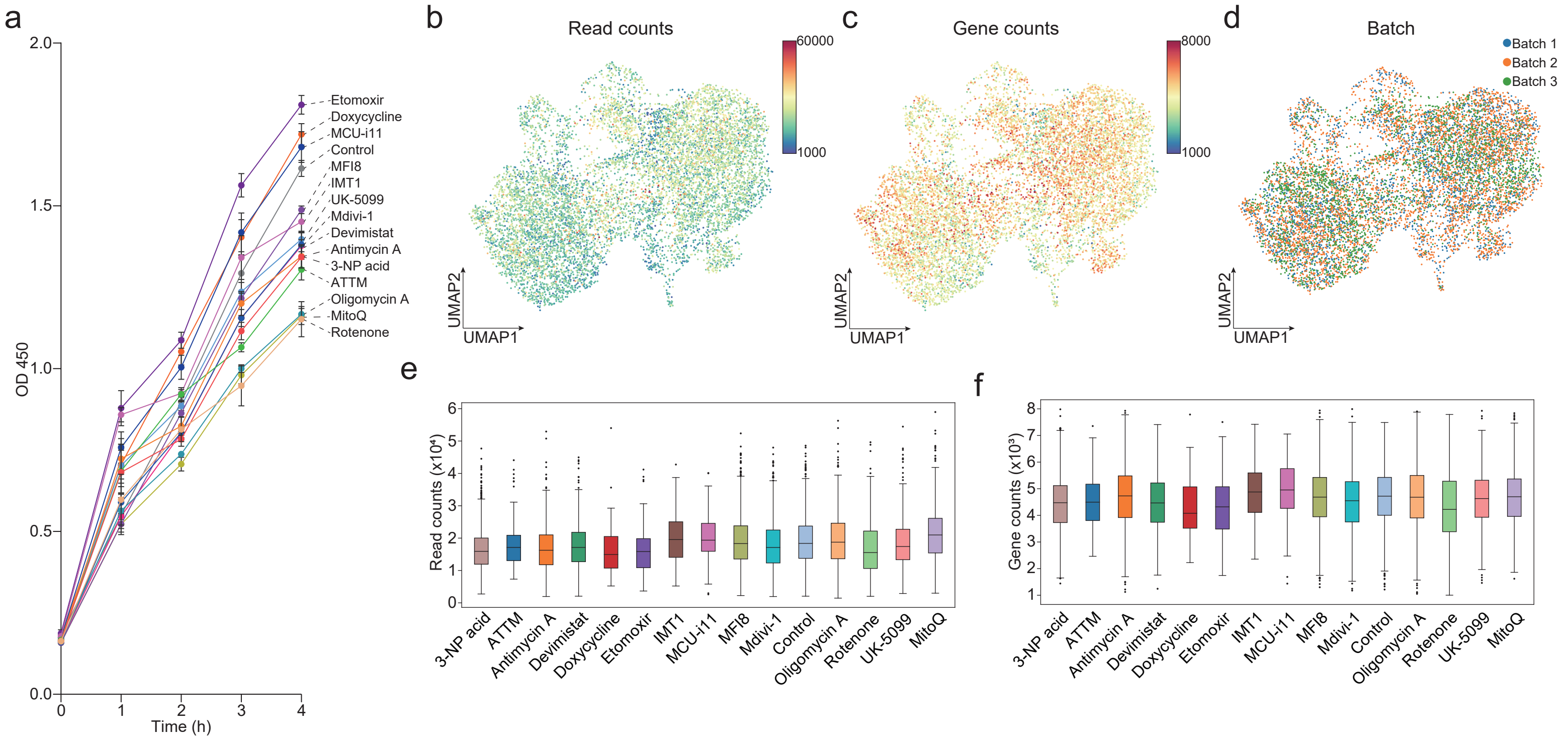

Supplementary Figure 2

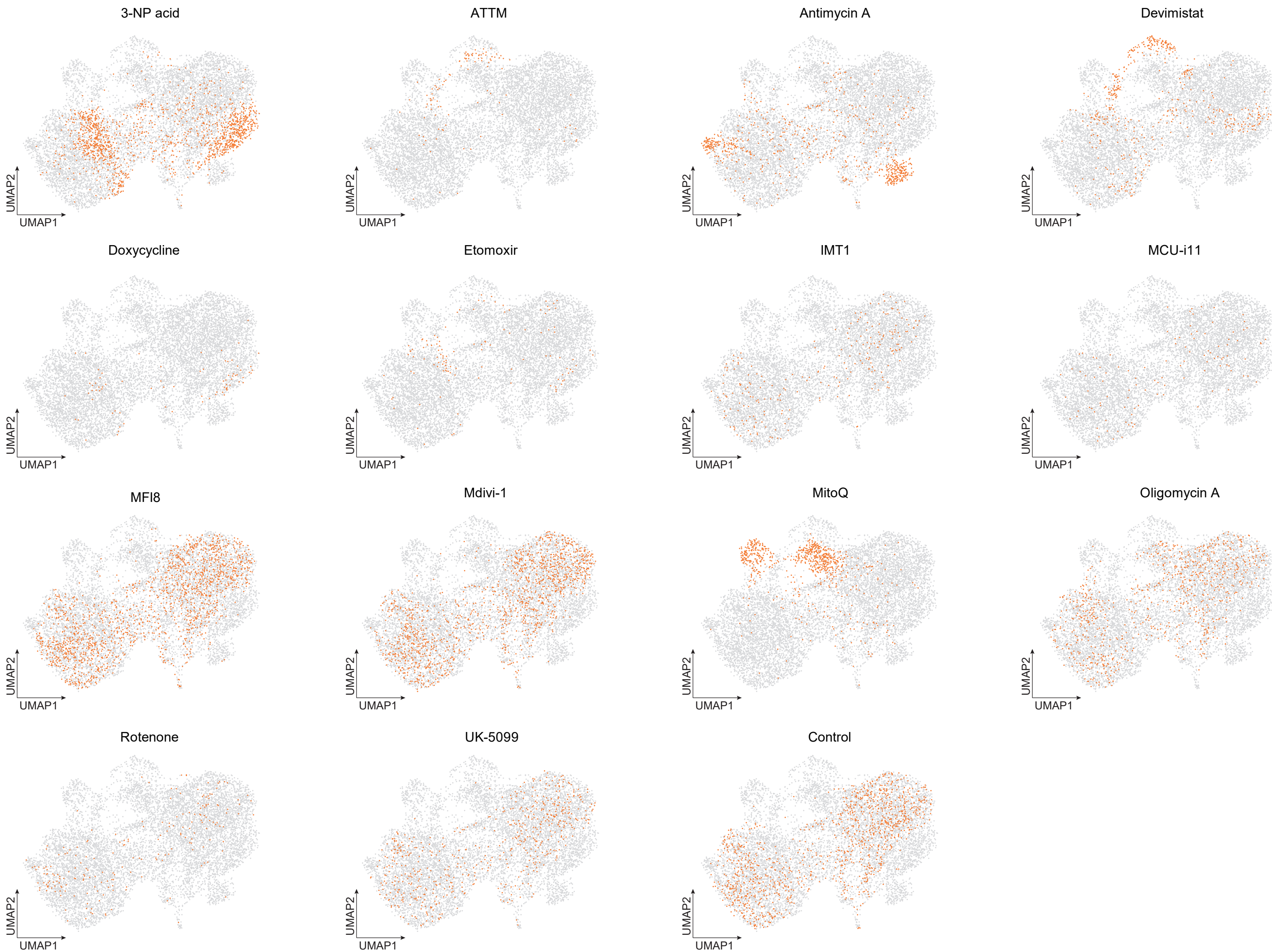

Supplementary Figure 3

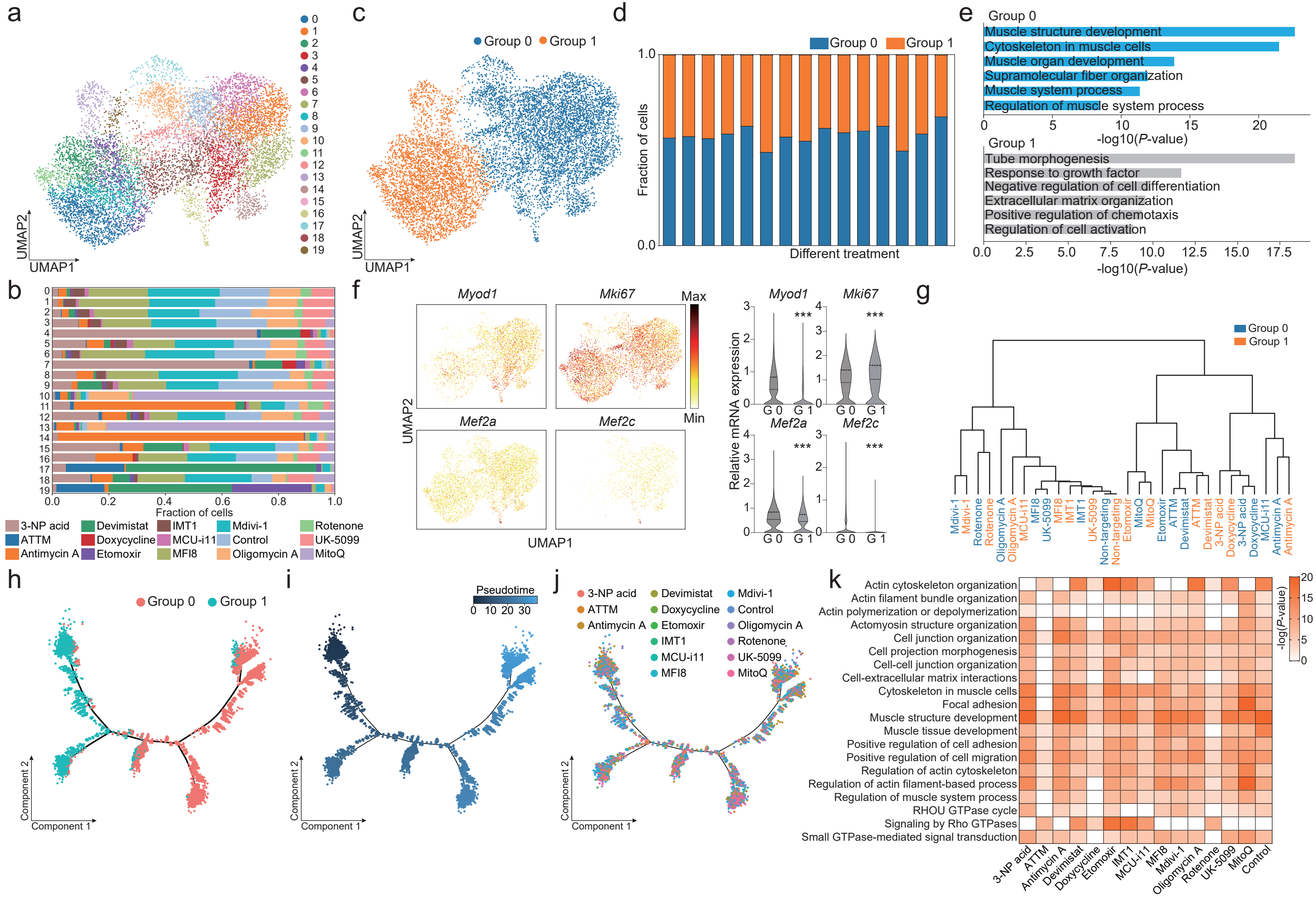

Supplementary Figure 4

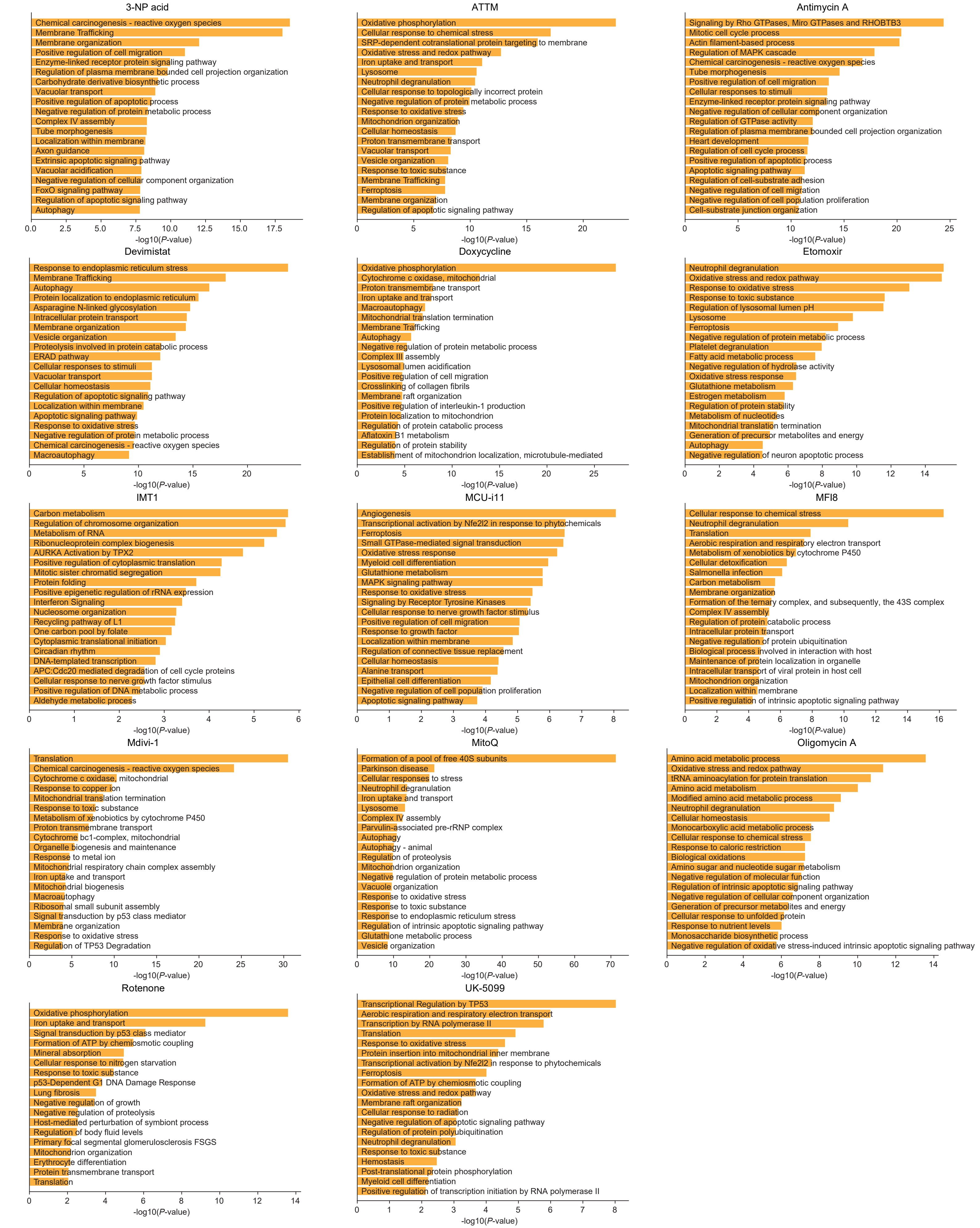

Supplementary Figure 5

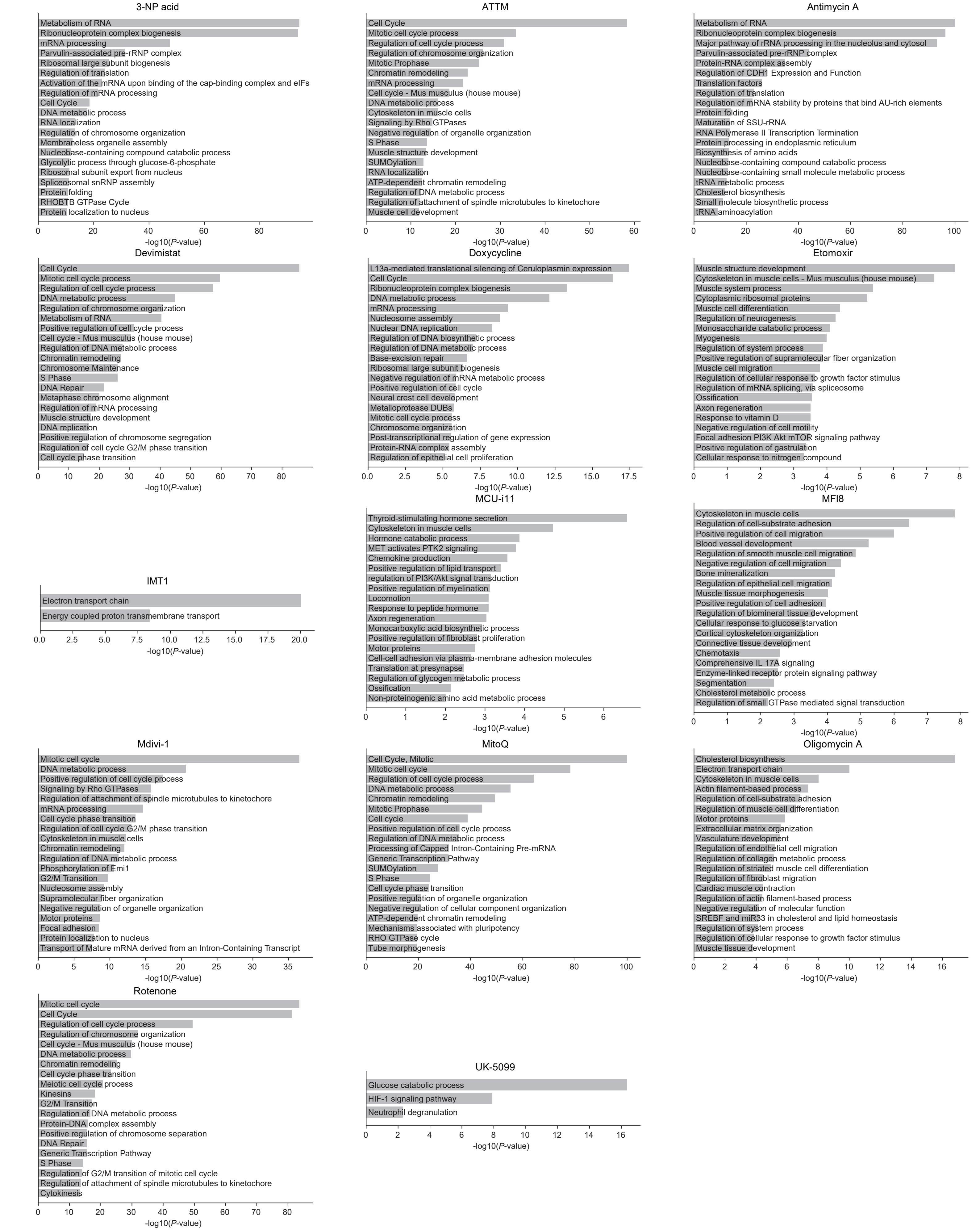

Supplementary Figure 6

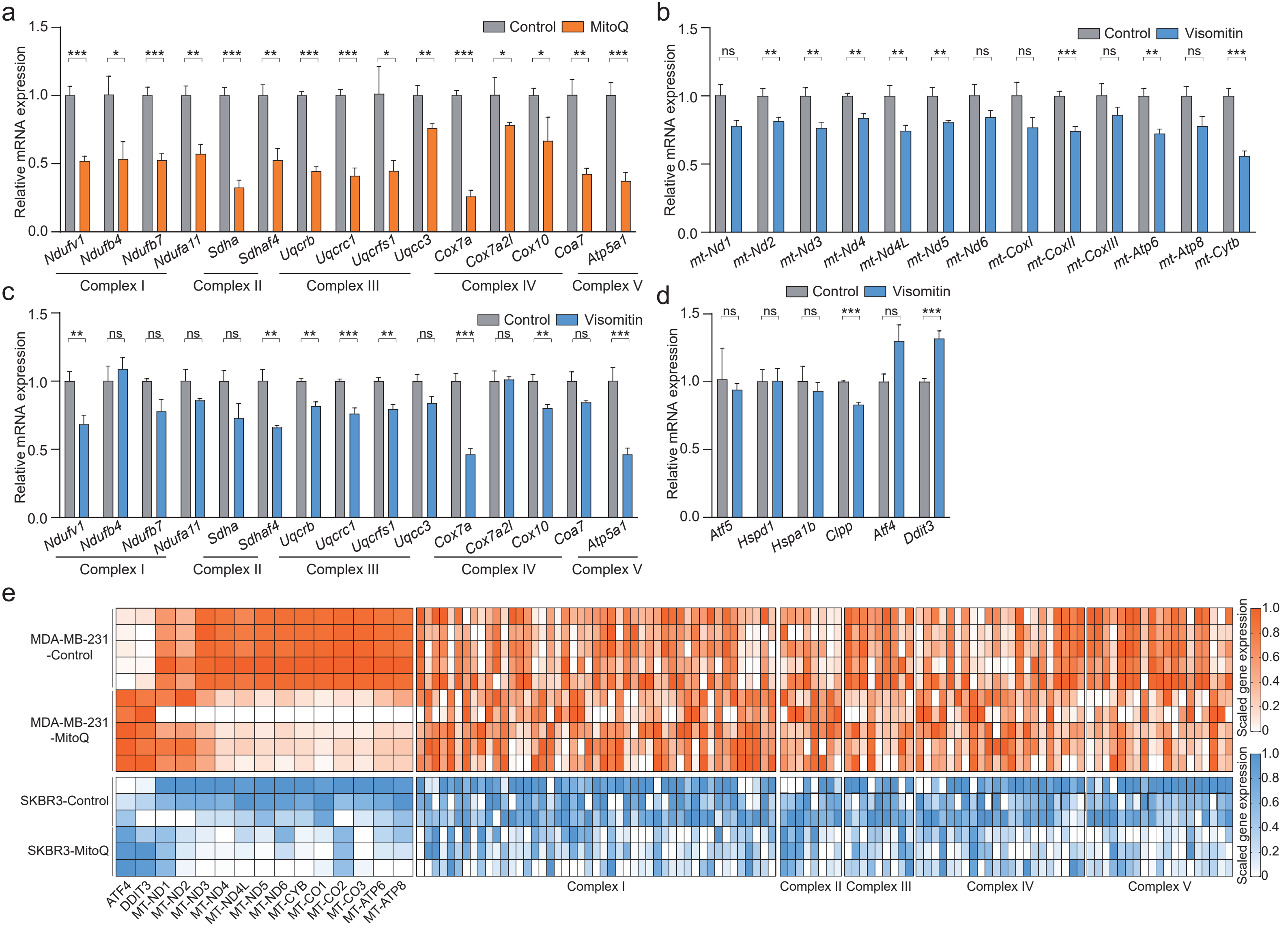

Supplementary Figure 7

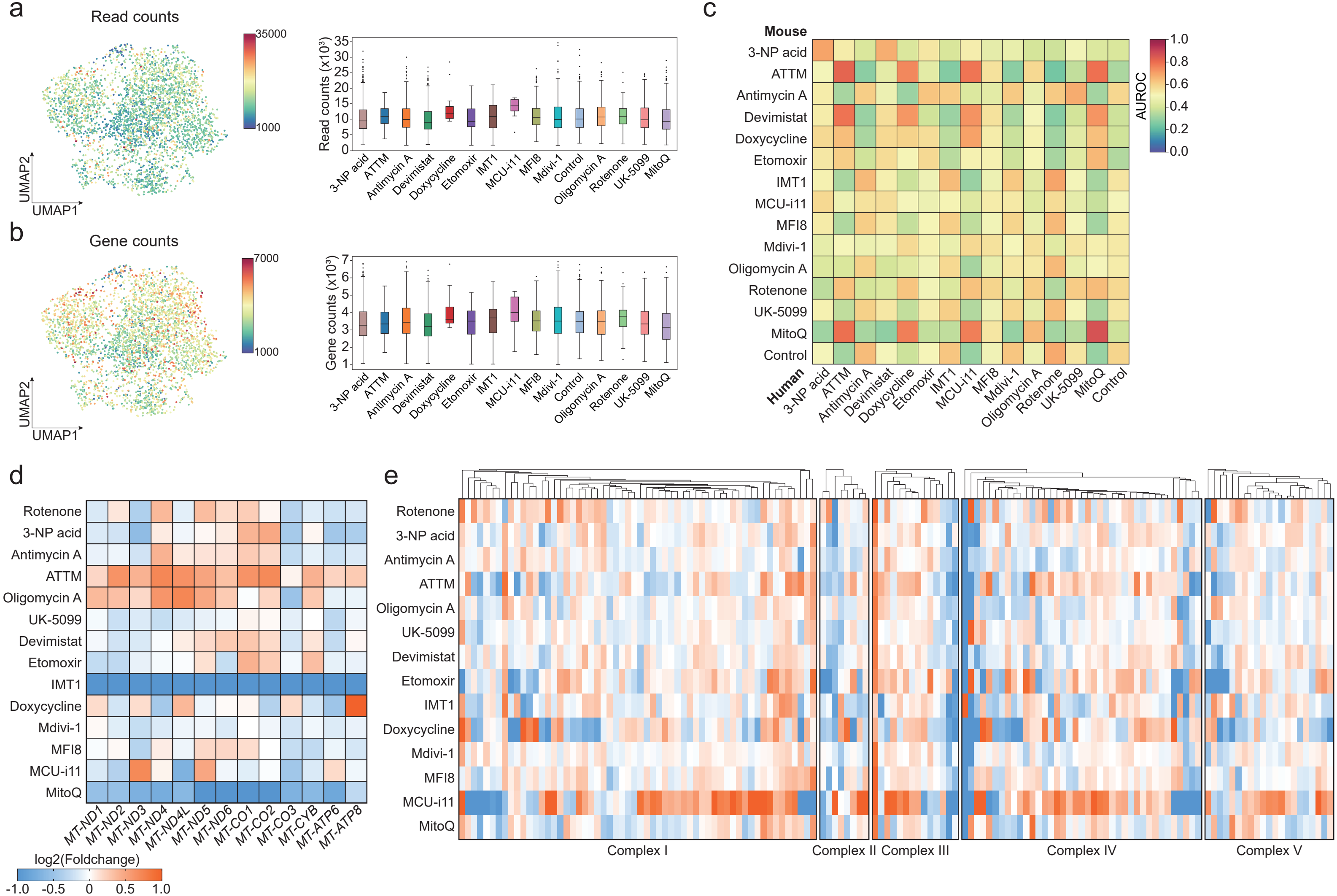

Supplement: Supplementary file 1 [file CPR-59-e70216-s002.pdf]
