# Supplementary material for: Dissection of Mitochondrial Function via Chemical Perturbation and Single‐Cell Profiling
Source: Cell Prolif. 2026 Apr 27:e70216. Online ahead of print. doi: 10.1111/cpr.70216 (PMC13325944; doi:10.1111/cpr.70216)

Supplementary Figure 1

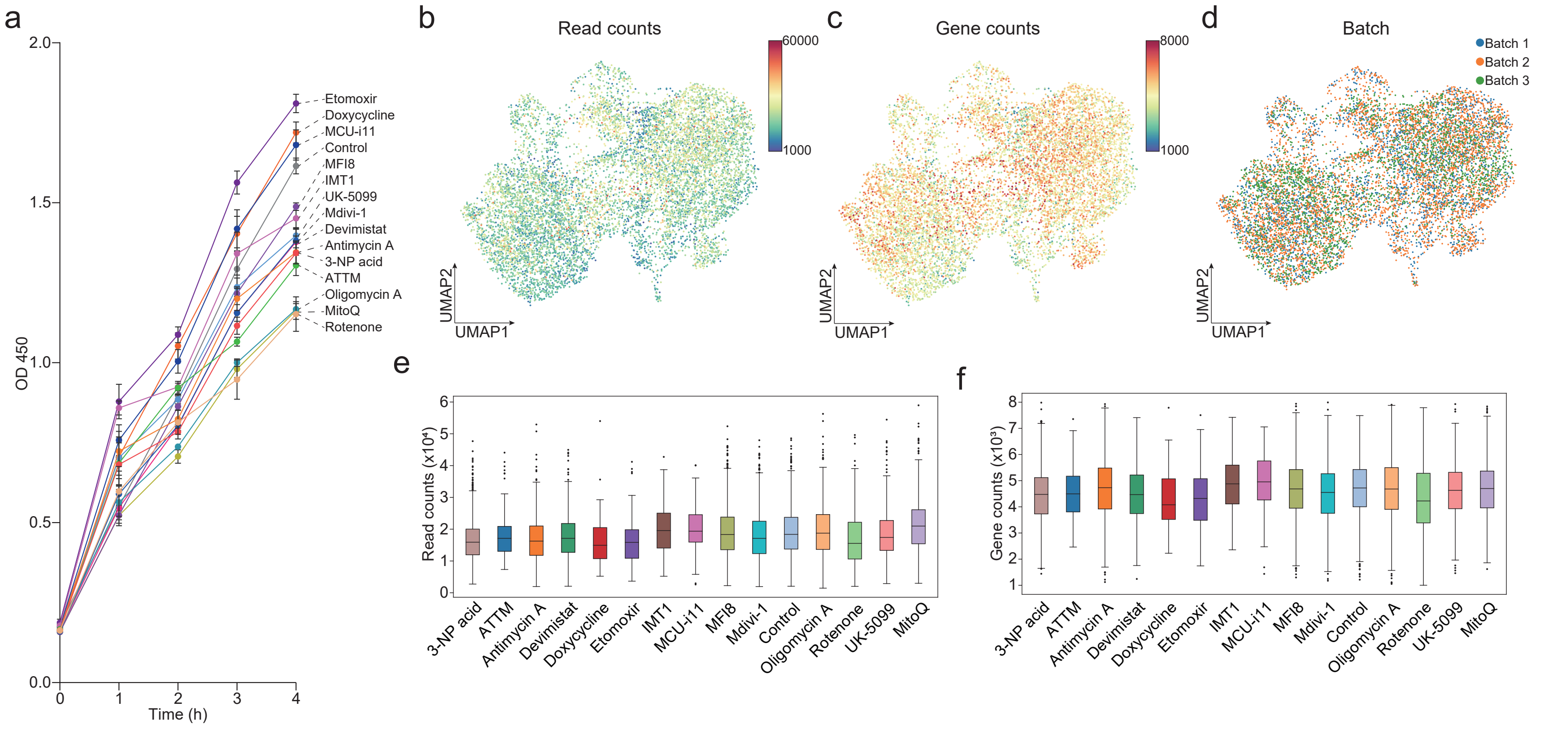

Supplementary Figure 2

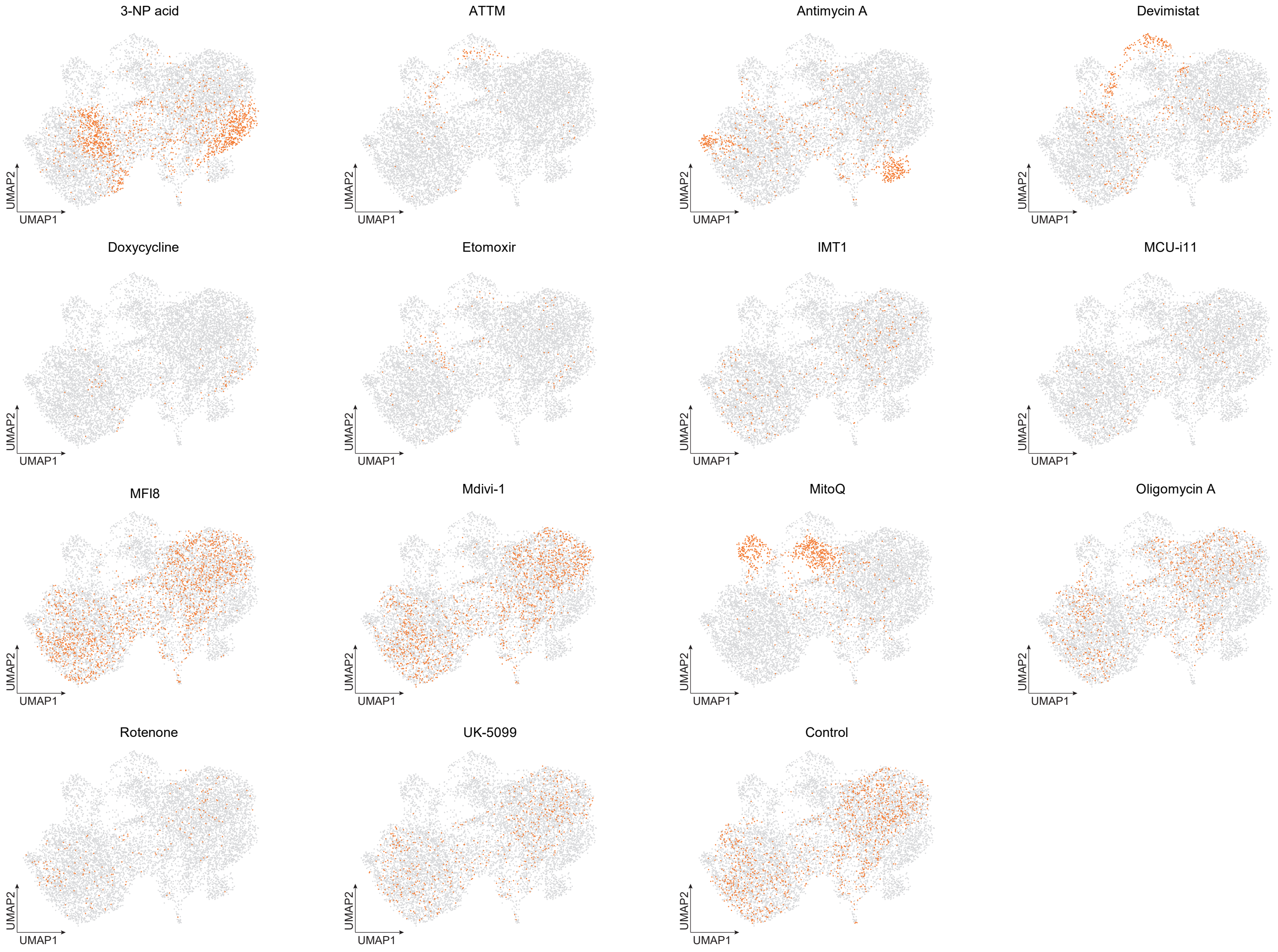

Supplementary Figure 3

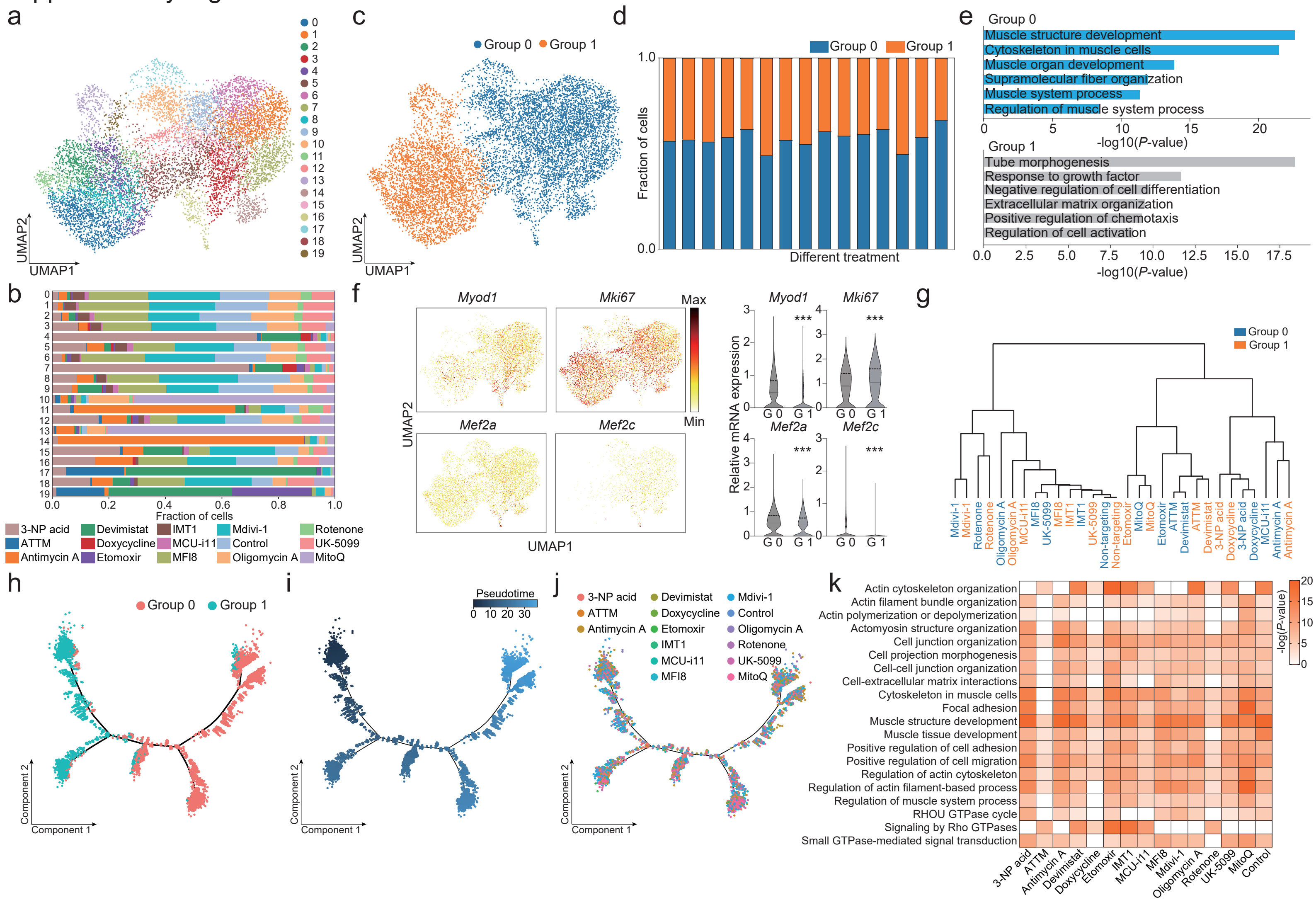

Supplementary Figure 4

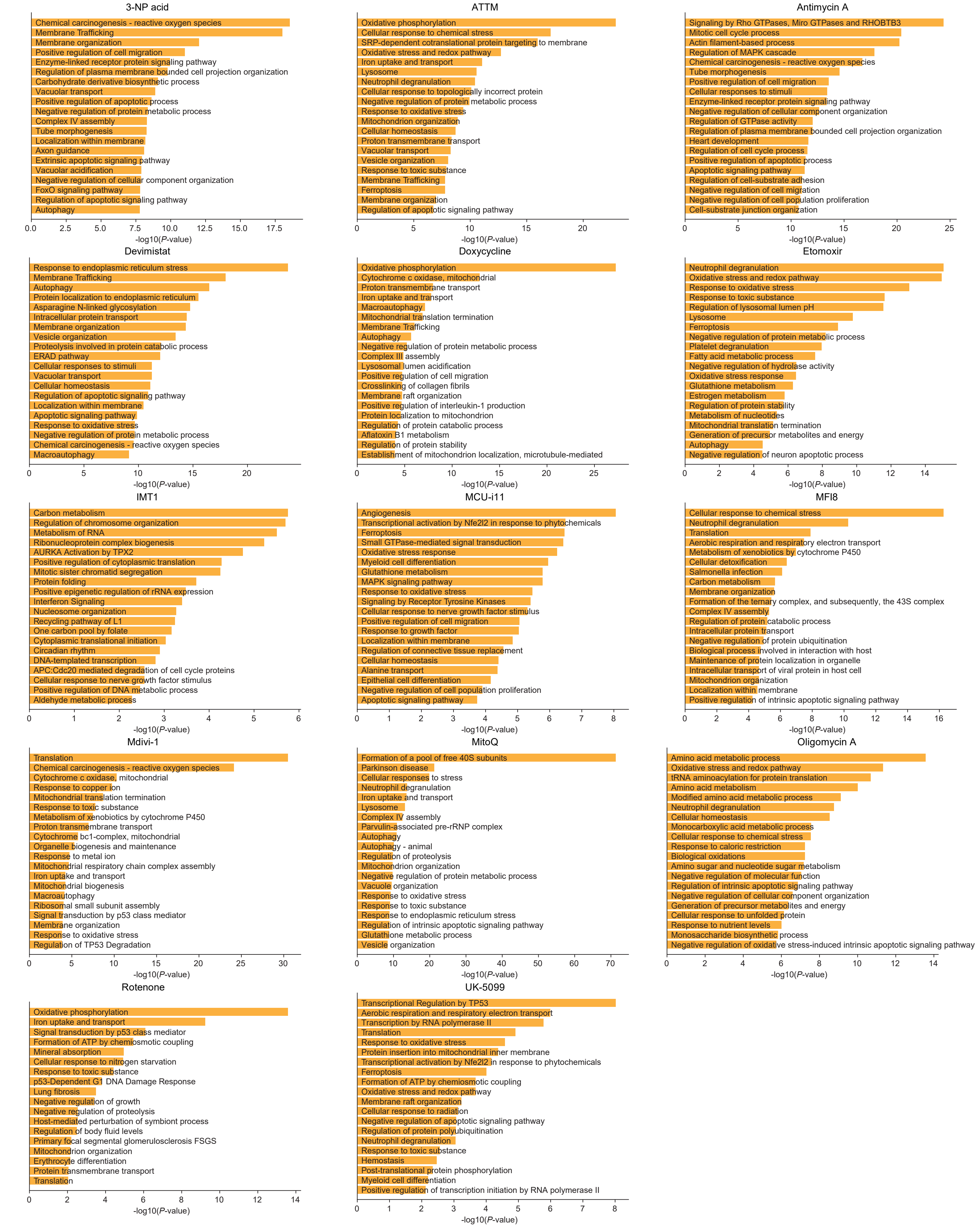

Supplementary Figure 5

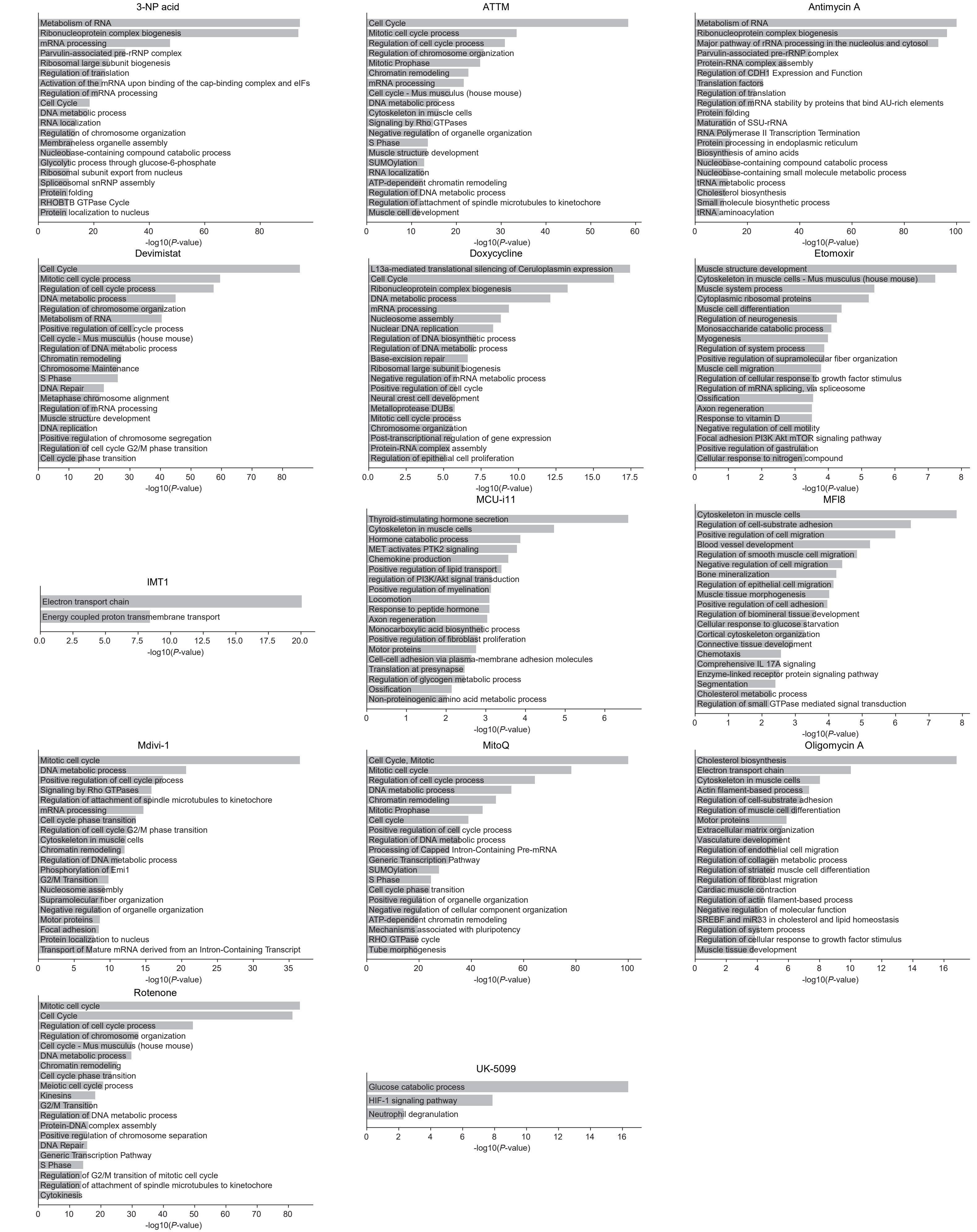

Supplementary Figure 6

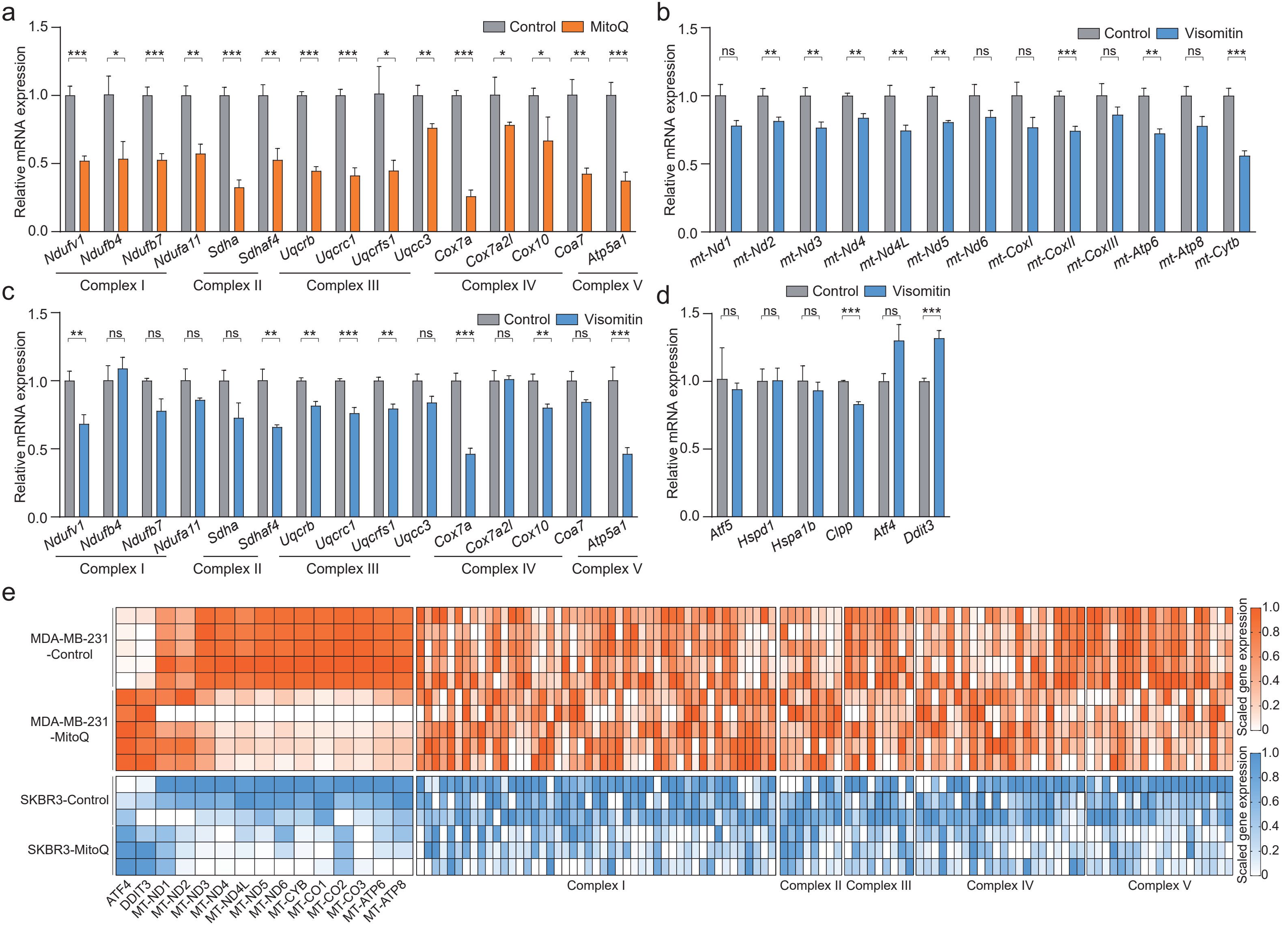

Supplementary Figure 7

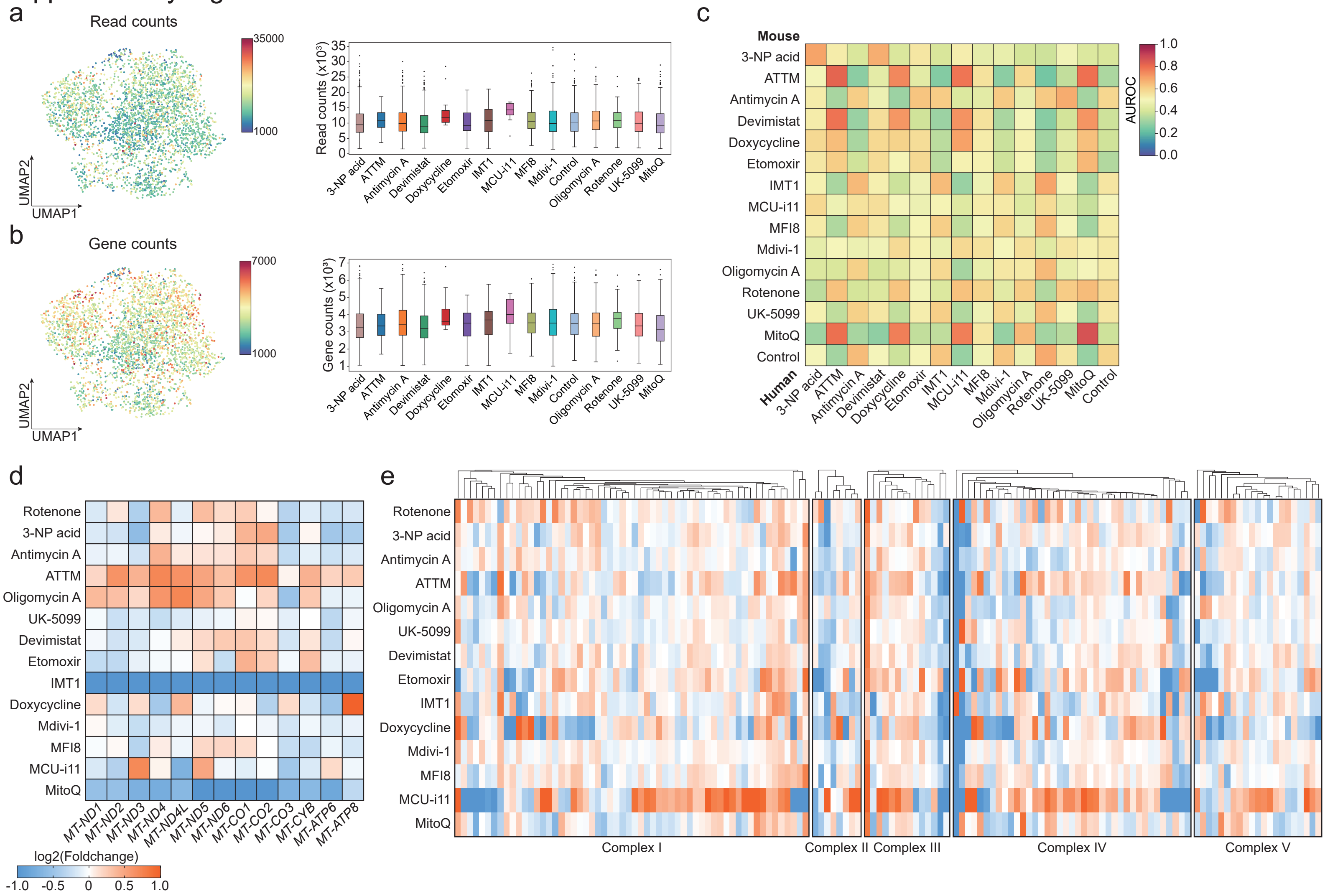

Supplement: Supplementary file 1 — Figure S1: Quality control of single‐cell data following different inhibitor treatments. (a) The line graph shows the results of the CCK8 assay measuring cell viability/proliferative capacity after 48 h of treatment with 14 inhibitors. Data are presented as mean ± SD. (b, c) UMAP plot shows the read counts and gene counts for each cell, with darker colours indicating higher values. (d) UMAP visualization shows the distribution of cells from three different batches, with each batch represented by a distinct colour. (e, f) Boxplot shows the distribution of read counts and gene counts across different treatment groups. Figure S2: UMAP plot shows the distribution of cells from 14 perturbation groups and control in mouse myoblasts, with each treatment highlighted in orange. Figure S3: Analysis of cellular heterogeneity. (a) UMAP plot shows the clustering results obtained using the Leiden algorithm. (b) Stacked bar plot shows the distribution of different treatment groups across each cluster. The horizontal axis represents the proportion, and the vertical axis represents the clusters. (c) UMAP plot of myoblast cells shows heterogeneity, with two distinct clusters. (d) Stacked bar plot shows the distribution of cells from each perturbation across Group 0 and Group 1. (e) Functional enrichment analysis of Group 0 and Group 1 based on genes upregulated in one group versus the other. (f) UMAP plot shows the expression levels of Myod1, Mki67, Mef2a, and Mef2c across cells, with darker colours indicating higher expression. The violin plots on the right provide a further statistical comparison of the expression of the genes shown on the left between the two groups. Solid lines indicate the mean, and dashed lines indicate the quartiles. *** p < 0.001 (two‐tailed Student's t‐test). (g) Dendrogram of hierarchical clustering shows the transcriptomic similarity of cells from each perturbation located in Group 0 and Group 1. Cells from Group 0 and Group 1 are shown in blue and orange, [file CPR-9999-e70216-s002.pdf]
